# Development of New SSR Markers for High-Throughput Analyses of Peach–Potato Aphid (*Myzus persicae* Sulzer)

**DOI:** 10.3390/insects16111156

**Published:** 2025-11-12

**Authors:** Jakub Vašek, Vladimíra Sedláková, Daniela Čílová, Martina Melounová, Ema Sichingerová, Petr Doležal, Ervín Hausvater, Petr Sedlák

**Affiliations:** 1 Department of Genetics and Breeding, Faculty of Agrobiology Food and Natural Resources, Czech University of Life Sciences Prague, Kamýcká 129, 165 00 Prague, Czech Republic; vasek@af.czu.cz (J.V.); sedlakova@af.czu.cz (V.S.); cilova@af.czu.cz (D.Č.); melounova@af.czu.cz (M.M.); 2 DIANA Biotechnologies, Průmyslová 596, 252 50 Vestec, Czech Republic; emasichin@gmail.com; 3 Department of Potato Protection, Potato Research Institute Havlíčkův Brod, Ltd., Dobrovského 2366, 580 01 Havlíčkův Brod, Czech Republic; dolezal@vubhb.cz (P.D.); hausvater@vubhb.cz (E.H.)

**Keywords:** *Myzus persicae*, peach–potato aphid, SSR, microsatellite, population analysis

## Abstract

The complex life cycle, high reproductive potential and ability to quickly develop resistance to insecticides are key factors contributing to the destructiveness of the peach–potato aphid (*Myzus persicae* Sulzer) among pest species. Monitoring its population dynamics at a large scale allows us to better understand *M. persicae* biology and take relevant measures for pest management. For this purpose, reliable molecular tools are needed. Based on the analysis of 128,362 microsatellite loci, we developed four multiplex assays including 49 comprehensively characterised SSR markers. Internal validation confirmed the species specificity and low genotyping error (*e_a_* = 0.8%, *e_l_* = 0.99%, *e_obs_* = 22.7%) of the assays. A total of 194 alleles were identified (mean = 4 alleles per locus, range = 2–8 alleles per locus) within a group of 365 aphid accessions collected in the Vysočina region (Czechia). The studied aphid population showed the typical characteristics expected of the species with clonal or partially clonal reproduction (heterozygote excess, negative F_IS_, moderate-to-high linkage disequilibrium (LD), and distortion of the H-W equilibrium for most of the loci), and did not exhibit any stratification on a spatiotemporal level. Owing to the high discriminatory power of the markers, we discovered that the population sample was founded upon a small number of fundatrices, as only five dominating lineages comprising over 70% of all accessions were identified. In conclusion, this study identified a significant number of new high-quality markers with the high discriminatory power necessary for revealing the population structure and dynamics of *M. persicae*, which holds considerable potential in both general biological and agricultural research.

## Simple Summary

The potato–peach aphid (*Myzus persicae* Sulzer) is one of the most destructive agricultural pests worldwide, causing economical losses through direct feeding and the transmission of plant viruses. It is a highly variable species, and knowledge of its population structure and dynamics, arising due to insecticide resistance and a change in host preferences, is beneficial for pest management and crop protection. The aim of this study is to present an innovative multiplexed panel of microsatellite markers for cost-effective, rapid and detailed population analysis of *M. persicae*. The marker set was designed based on the latest chromosomally assembled genome to cover equally and relatively densely all of the species’ chromosomes. Each marker was carefully verified on a broad sample of individuals and incorporated into the panel based on the detected population variability and reproducibility. The primers were highly specific to *M. persicae*; however, partial transferability to *Phorodon humuli* was found. This transferability enables wider utilisation and improves the informational value compared to previously used markers. The markers were verified for their ability to detect general population diversity, showing promise for future research on the population stability of the species, rearrangements in clones, and population and individual changes related to sex formation and sexual reproduction.

## 1. Introduction

The peach–potato aphid (*Myzus persicae* Sulzer; Hemiptera: Aphididae) is a significant pest for a broad range of crops, vegetables and ornamental plants worldwide. This species causes immense losses in the yield and quality of crops through direct feeding and the transmission of more than 100 plant virus species [1]. In Czechia, it is recognised as a serious vector of several viruses, posing a particular threat to seed potato production.

The standard karyotype of *M. persicae* is 2n = 12; however, the number of chromosomes ranges from 12 to 17 because of various autosomal rearrangements [2,3,4]. The most frequent variant of the female karyotype 2n = 12 is represented by a reciprocal translocation between chromosomes 1 and 3 [5]. The translocation is known to be linked with organophosphate and carbamate resistance [6]. The sex chromosome X appears structurally highly conserved [7] compared to the autosomes. It is the longest member of the karyotype, generally exhibits a down-regulated expression of genes, and contains some genes specifically expressed only in males [4,8].

The species exhibits holocyclic reproduction in temperate zones across all continents; in warmer climatic conditions, the anholocycly prevails [9]. The holocycly is represented by a series of generations produced by apomictic parthenogenesis, followed by one sexual generation. Occurring and mating typically in autumn, males (2n = 11 + X0) and sexual females (2n = 11 + XX) produce fertilised overwintering eggs on *Prunus persica* L. plants. After diapause in spring, parthenogenetic females hatch and produce flying offspring, which attack secondary host plants and reproduce by parthenogenesis [10,11].

The development of sexual generation is driven by temperature and photoperiodism—the typical conditions are 10 h of light and 14 h of darkness at 15 °C [9,12,13]—when a titre changes in the juvenile hormone. Whilst a high titre level in short-night periods supports parthenogeny, a low titre level in long-night periods signifies the development of sexual reproduction [14]. Then, parthenogenetic females, under the effect of a programmed loss of one random chromosome X (arrhenotoky), rarely produce males [15].

The predominant parthenogenesis should conserve allelic diversity and reduce genotype diversity of loci, as was notably observed in *Rhopalosiphum padi* [16] and *Acyrthosiphon pisum* [17]. However, despite this expectation, *M. persicae* is characterised by considerable intra- and inter-population genetic diversity [18]. The genetic diversity of aphids has been extensively studied using various molecular methods. Although contemporary strategies based on whole-genome sequencing [4,19] are available, they are prohibitively expensive and sophisticated and, thus, are not considered suitable for aphid diversity studies in which analysing large numbers of individuals is necessary. In contrast, as codominant and hypervariable microsatellites [20] are time- and cost-effective, they have considerable potential for analysing most species of aphids [16,17]. To date, twenty-nine microsatellite markers of *M. persicae* have been identified [21,22,23,24], and four of them are localised on chromosome X. Such a low number of markers represents only a limited part of the total genetic diversity of the species; however, recent whole-genome sequencing projects on *M. persicae* [4,19,25] have made a broad spectrum of microsatellite loci available, improving not only the multiplexing but also the capacity of analytical assays.

The present research focussed on the design and optimisation of multiplex assays covering a significantly higher number of variable microsatellite markers on all the chromosomes of *M. persicae*. The main objective of this work is to present the results of the development of these assays and a preliminary analysis of the experimental population samples of *M. persicae* collected in the main potato growing area of Czechia in summer 2024.

## 2. Materials and Methods

### 2.1. Bioinformatic Analysis

The reference aphid genome (GCF_001856785.1) [26] was used as a primary source for microsatellite loci mining. Subsequently, when the chromosome-level assembly of aphid genome (GCA_029232275.1) became available [25], information about the chromosomal position of selected markers was implemented. Mining was conducted with the help of GMATA v2.0 [27] wherein only perfect microsatellites with a motif length of 2–6 nt and a minimum of 5 repetitions were searched. The obtained data files were further processed and analysed via R v4.3.3 [28] with additional packages, including dplyr v1.1.4 [29], tidyr v1.3.1 [30] and purrr v1.0.2 [31] for data manipulation and transformation tasks; jsonlite v1.8.8 [32] for uploading JSON files; stringr v1.5.1 [33] for text string searching and formatting; ggplot2 v3.5.1 [34], patchwork v1.3.0 [35] and ggrepel v0.9.6 [36] for graphical visualisation; flextable v0.9.10 [37] for tabular output; and knitr v1.50 [38] and rmarkdown v2.29 [39] for textual output.

An important step of data analysis was the removal of unsuitable or low-quality loci. Therefore, only tri- to hexanucleotide microsatellites with at least 7 repetitions were selected. Moreover, the selected loci were thoroughly tested using a slightly modified version of the R script developed during our previous research [40]. The R script enables the analysis of the results output by Basic Local Alignment Search Tool-Nucleotide (BLASTN) v.2.14.0+ [41] per locus for quality evaluation based on predefined criteria (e.g., number of sequences with high query cover or total count of sequences producing significant alignment). Finally, new primers were designed for the remaining loci via Primer3 v4.1 [42] and OligoEvaluator (an online web tool provided by Merck available at https://www.oligoevaluator.com/LoginServlet (accessed on 27 October 2025)).

### 2.2. Sampling Material and DNA Extraction

Samples of aphids were collected using yellow pan traps with a common detergent solution during the growing season of 2024 at potato plantations located in Humpolec (49°37′47.9″ N, 15°35′22.1″ E) and Valečov (49°38′33.6″ N, 15°29′45.0″ E), both in the Vysočina region. The traps were placed on the top of plants in the experimental potato plots of the Potato Research Institute Havlíčkův Brod. Aphids were collected three times per week and sorted. Individuals of the collected aphid species were subsequently washed in 50% ethanol and water and separately placed in 0.5 mL tubes. Besides *Myzus persicae* (*N* = 450), several individuals of *Aphis nasturtii* (*N* = 7), *Phorodon humuli* (*N* = 6) and *Aphis fabae* (*N* = 7) were also collected to test the specificity of the developed markers. The aphids were morphologically identified by entomologists at the Potato Research Institute Havlíčkův Brod based on the standards EPPO PP1/230-1 “Aphids on potato” [43], and detailed descriptions were uploaded to the database Encyclop‘Aphid (INRA, Paris, France, https://www6.inra.fr/encyclopedie-pucerons (accessed on 27 October 2025)). The samples were stored in a fridge (−20 °C) for nucleic acid extractions.

The nucleic acids of individual samples were extracted using TRI Reagent (Sigma, Aachen, Germany). The conditions were optimised as follows: The aphid individuals were homogenised in 100 μL of the TRI Reagent solution using a glass rod. After 10 min of incubation, 20 μL of stabilised chloroform was added, and the solution was thoroughly vortexed and centrifuged at 12,000 rfc for 15 min (Centrifuge 5430-R, Eppendorf, Hamburg, Germany). The water phase was transferred to a new tube for RNA extraction. A total of 30 μL of absolute ethanol was added to the organic phase to precipitate DNA. The solution was mixed by inversion, incubated for 5 min and centrifuged at 3200 rfc for 6 min (Centrifuge 5430-R). The supernatant was removed by pipetting, and the pellet was dissolved in 100 μL of the wash solution (0.1 M sodium citrate solution in 10% ethanol, pH 8.5). The samples were incubated for 30 min and centrifuged, and the wash step was repeated. After removing the wash solution, the pellet was washed with 150 μL of 75% ethanol, incubated for 20 min and centrifuged. The pellet was dried and dissolved in 20 μL of the AE elution buffer (Qiagen, Hilden, Germany). The undiluted DNA samples were directly used for microsatellite analysis.

### 2.3. Quantification by Digital PCR

Precise gDNA quantification of the samples selected for the optimisation of capillary electrophoresis was conducted via droplet digital PCR (ddPCR) using the QX200 Droplet Digital PCR System (Bio-Rad, Hercules, CA, USA). The ddPCR reaction mixture with a total volume of 40 μL consisted of 1 μL of unquantified DNA, 0.2 μM of a pair of primers, and 1× QX200 EvaGreen ddPCR SuperMix (Bio-Rad, Hercules, CA, USA). The two-step PCR comprised 1 cycle of initial denaturation at 95 °C for 2 min, 40 cycles of denaturation at 95 °C for 30 s, annealing + elongation steps at 60 °C for 1 min, 1 cycle of cooling at 4 °C for 5 min, and 1 cycle of droplet stabilisation at 90 °C for 5 min. All steps used a 2 °C/s ramp rate. The obtained data were processed and analysed in QuantaSoft v1.7.4 (Bio-Rad, Hercules, CA, USA).

### 2.4. PCR and Initial Marker Screening

The PCR mix with a total volume of 10 μL contained 0.25 ng of genomic DNA, 0.2 μM of a pair of primers, and 1× Multiplex PCR Master Mix Plus (Qiagen, Hilden, Germany). The amplification conditions were as follows: 1× pre-denaturation step at 95 °C for 10 min, 35× repeated steps of denaturation at 95 °C for 30 s, annealing at 60 °C for 1.5 min, and elongation at 72 °C for 1 min. The final elongation step was run at 72 °C for 10 min. All samples were amplified using a C-1000 (Bio-Rad, Hercules, CA, USA) or T-gradient Thermo (Analytic Jena, Jena, Germany) thermocycler. The obtained PCR amplicons were electrophoretically separated in 2% (*w*/*v*) agarose gel. The first round of PCR and amplicon separation was conducted using three selected samples per candidate marker to verify the expected size of the amplicons. The second round of PCR amplification and electrophoretic separation in 4% (*w*/*v*) agarose gel used nine samples per marker to identify polymorphic loci, and only loci with a clear profile and one or two distinct bands per sample were subjected to Sanger sequencing as an additional quality control step.

### 2.5. Amplicon Sequencing

The PCR reaction and amplification conditions were the same as previously described, with the exception that the amplicons were separated on 1% agarose gel, excised by a clean scalpel, and purified with the GenJET Gel Extraction Kit (Thermo Fisher Scientific, Waltham, MA, USA). Only one minor change was made for small amplicons (100–150 bp), where locus-specific primers with M13 tails, instead of the original primers, were used (F primer tail—5′ TGTAAAACGACGGCCAGT 3′ + locus-specific sequence, R primer tail—5′ CAGGAAACAGCTATGACC 3′ + locus-specific sequence). The purified DNA was quantified using a UV nanophotometer S111107AW (Implen, Munich, Germany), and the concentration was first adjusted according to the requirements of the sequencing service provider (EuroFins Genomics Germany GmbH, Ebersberg, Germany) and then bidirectionally sequenced. The obtained raw sequences were checked using Sequencing Analysis Software v5.2 (Thermo Fisher Scientific, Waltham, MA, USA) and assembled using BioEdit v7.0.9.0 software [44] and MUSCLE v3.8 [45].

### 2.6. Multiplex PCR and Capillary Electrophoresis

The PCR for capillary electrophoresis (CE) was slightly modified from the PCR used for initial screening. The DNA amount was lowered to 40 pg in the case of “optimisation” samples quantified via ddPCR or was adjusted to a volume of 2 μL in the case of unquantified “population” samples. The primers were pooled into four multiplex assays with the use of the Primer Pooler program [46], their concentration was optimised, and each forward primer was labelled with a fluorescent dye (Table 1). Moreover, the final elongation step of the PCR program was set to 60 °C for 1 h 20 min and the number of cycles was changed from 35 to 29. Further, one microliter of the PCR product was diluted in 12 μL Hi-Di formamide (Thermo Fisher Scientific, Waltham, MA, USA) with 0.25 μL GeneScan LIZ600 Dye Size Standard v2.0 (Thermo Fisher Scientific, Waltham, MA, USA) and denaturated at 95 °C for 5 min. This was followed by separation of the samples using the ABI PRISM 310 Genetic Analyser (Thermo Fisher Scientific, Waltham, MA, USA), with 8 s injection at 1.5 kV and a run time of 25 min at 15 kV.

To correct for underlying fragment size variability that arose between individual runs and between plates, an initial allelic ladder per each plate (Figure S1) was included. The ladder was carefully prepared from a mixture of single-locus amplicons derived from reference samples of *M. persicae*. The possibility of contaminations was checked using non-template controls subjected to the same treatment as the analysed samples. Fragment analysis and allele identification were conducted using GeneMapper v4.1. software (Thermo Fisher Scientific, Waltham, MA, USA).

### 2.7. Genotyping Error and Missing Data Analysis

To estimate genotyping error, 22 genotypes (~5% of *Myzus persicae* samples) were randomly selected using the random sampling function sample() in R. These samples served as technical replicates in situation where only one replicate per sample was amplified due to the very limited amount of DNA available. The amplification conditions and data analysis were the same as for the reference genotypes. The calculation of error rate followed the methodology proposed by Taberlet’s research group [47], utilising three metrics—mean error rate per allele (*e_a_*), mean error rate per locus (*e_l_*), and error rate per multilocus genotype (*e_obs_*). The dataset was further checked for the occurrence and distribution of missing values by applying two parameters—proportion of missing samples per marker and proportion of missing markers per sample.

### 2.8. Population Analysis

A preliminary population analysis of the samples collected from the Vysočina region was conducted to determine the informational value of the newly developed markers for diversity studies. A purified dataset was transformed into a “genind” object using the adegenet package v2.1.11 [48], as this was the most suitable data format recognised by many population genetics packages in R. This package was used to calculate basic descriptive parameters, including number of alleles per locus (N*_a_*), expected heterozygosity (H_Exp_), and observed heterozygosity (H_Obs_). Other statistics, namely inbreeding coefficients (F_IS_ and F_ST_) and Hardy–Weinberg equilibrium (HWE) tested via the $\chi^{2}$-test and exact test (set as 10,000 Monte Carlo permutations) for each locus, were obtained using the pegas v1.3 [49] and hierfstat v0.5-11 [50] packages. The HWE assumption on a global scale was statistically tested using paired Welch’s test and a nonparametric paired Wilcoxon signed-rank test, because the exploratory data analysis (EDA) revealed potential violation of model assumptions required for the application of parametric tests. Gaussian distribution was checked visually and using the Shapiro–Wilk test. Homogeneity of variance was checked with Bartlett’s test. All statistical tests were judged at a significance level α = 0.05.

Diversity indices, including λ (Simpson’s diversity index), G (Taylor’s index), H (Shannon–Weaver index), evenness (E), and the global and paired standardized association indices (${\overset{´}{r}}_{D}$), which is used to estimate whether loci are in linkage disequilibrium (LD), were obtained using the poppr package v2.9.6 [51,52].

The population structure on a spatiotemporal level was explored via Principal Coordinate Analysis (PCoA) available in the ade4 package v1.7-23 [53] and the minimum spanning network (MSN) available in the poppr package. For the PCoA analysis, the matrix of alleles counts was transformed into a binary matrix, which was then transformed into a distance matrix based on the Jaccard index. MSN was based on Bruvo’s distance [54] which was specifically developed for microsatellite markers based on the assumptions of a stepwise mutational model.

Furthermore, as the complex life cycle of aphids leads to partially clonal populations, most analyses were performed twice, with and without clone correction (i.e., with and without the removal of samples with duplicated genotypes). This enabled the observation of the impact of clonal reproduction on the values of commonly used population descriptive parameters.

## 3. Results

### 3.1. Bioinformatic Analysis and Marker Development

**Figure 1:**
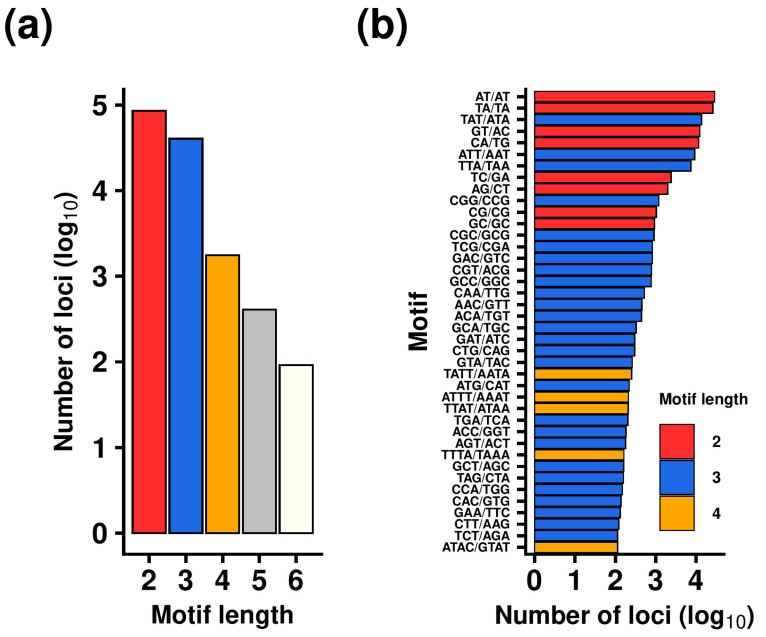
Distribution of SSR loci by motif length (**a**) and motif (**b**) across *M. persicae* genome.

A total of 128,362 perfect microsatellites, each comprising five or more repeat units, were identified within the aphid genome. The number of available loci declined with a longer motif, and a sharp drop in the number of tetra- to hexanucleotide microsatellites was observed, as these groups represented only ~1.8% of all detected loci (Figure 1a). On the contrary, di- and trinucleotide microsatellites clearly dominated, accounting for ~66.7% and ~31.5% of the total, respectively. The analysis showed that the average number of repeats was between 7 and 8 (7.44) according to the mean, or 6 according to the median. The most frequent microsatellite motifs were AT, TA and TAT (Figure 1b).

**Figure 2:**
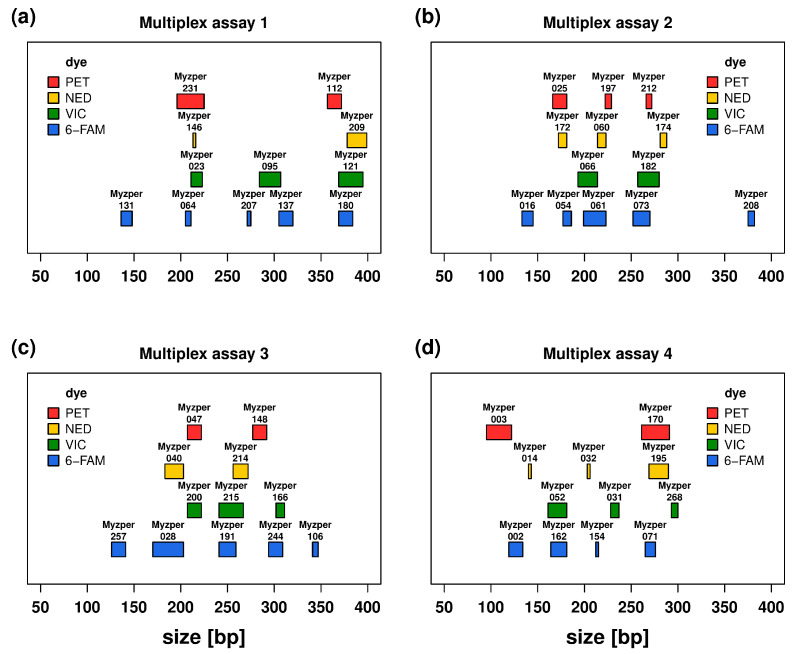
Graphical representation of multiplex assays (**a**–**d**) where polygon width reflects the size range of the detected alleles for a given locus.

**Figure 3:**
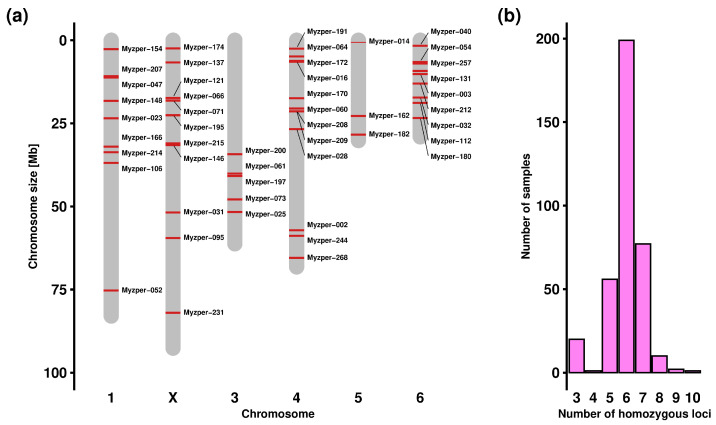
Chromosomal localisation of SSR markers (**a**) and distribution of homozygous loci per sample for X-linked markers (**b**).

Although the initial number of available loci was high, the application of stringent criteria and several rounds of quality filtering led to the identification of a substantially lower number of candidate loci (539 tri-, 7 tetra- and 2 pentanucleotide SSRs), and there were only 274 loci whose designed primers fulfilled all set parameters (T_m_ ≥ 60 °C, thermodynamic oligo alignment, and a zero value for 3′ self-complementarity, pair-complementarity and primer hairpin). The wet lab screening with two rounds of agarose gel electrophoresis retained 224 and 115 loci, respectively, and specificity verification via Sanger sequencing (61) and capillary electrophoresis separation (49) further reduced the set of available markers (the number in round brackets shows the number of retained loci after a given analysis). The remaining markers were split into four optimised multiplexes, with 12, 13, 12 and 12 markers (Figure 2). These newly developed markers are distributed across all *M. persicae* chromosomes with a mean of 8 markers and a range of 3 to 11 markers per chromosome (Table 2 and Figure 3a).

### 3.2. Genotyping Error and Missing Data Analysis

The least stringent metric—the allelic error rate (*e_a_*)—reached a mean of 1.65% (median = 0), with a range of 0 to 21.4% across all markers, while the genotyping error (*e_l_*) was higher (mean = 2.56%, median = 0%), ranging from 0 to 42.9% across all markers. Most of the markers appeared to be reliable according to the median values, but some loci, namely Myzper-131, Myzper-182 and Myzper-180 (Table S1), were highly prone to error. When these loci were excluded in a stepwise manner, the mean error rate per allele (*e_a_*) gradually dropped from 1.65% to 1.22% and from 0.98% to 0.8%. Similarly, the mean error rate per locus (*e_l_*) gradually dropped from 2.56% to 1.72% and from 1.28% to 0.99%. The exclusion of these error-prone loci had the most notable impact on the estimation of the multilocus genotyping error (*e_obs_*) when whole-genome profiles were evaluated. The value of *e_obs_* was initially relatively high, at 59%, but the exclusion of the three error-prone loci lowered the value to 22.7%. Two more loci, Myzper-052 and Myzer-268, showed an elevated error rate (Table S1) and were initially considered for exclusion, but they were later retained as the error rate per multilocus genotype was not substantially improved with their exclusion (*e_obs_* = 18.18%).

The exclusion of error-prone markers was conducted alongside the removal of low-quality samples. Missing data analysis revealed that the number of missing samples per marker ranged between 5% and 23.4%, with mean = 11.9% (median = 10.9%), while the number of missing markers per sample ranged between 0% and 100%, with mean = 11.9% (median = 0%). Only samples with no more than four missing markers (maximum of ~8.7% of missing markers per sample) were retained. Dataset filtering steps led to substantial quality improvement: the number of missing samples per marker dropped between 0% and 13.4%, with mean = ~0.94% (median = 0%), and the number of missing markers per sample dropped between 0% and 8.7%, with mean = ~0.56% (median = 0%). As expected, the filtering process led to the exclusion of all accessions of other aphid species, except for one *P. humuli* representative (probably misclassified as *M. persicae*). A specificity test across aphid species showed low marker transferability for *A. fabae* and *A. nasturtii*, with only two amplifying markers (4.1%), and high transferability for *P. humuli*, with 33 (67.3%) amplifying markers for more than one sample (Table S2).

### 3.3. Population Analysis

As a component of the comprehensive population analysis, sex determination was performed using molecular markers. For this purpose, 11 new X-linked SSR loci (Table 2, Figure 3a) were identified via in silico analysis. Sex determination was based on the concept of exploiting the differences in the number of X chromosomal copies between sexes, where females possess two copies of the X chromosome whereas males possess only one copy. Consequently, hemizygous males should resemble homozygotes for all X-linked markers from an analytical point of view. The frequency analysis revealed the distribution of homozygous loci per sample, with a mean of 6 and a range of 3 to 10 homozygous loci (Figure 3b). These findings led to the conclusion that all accessions are females.

**Figure 4:**
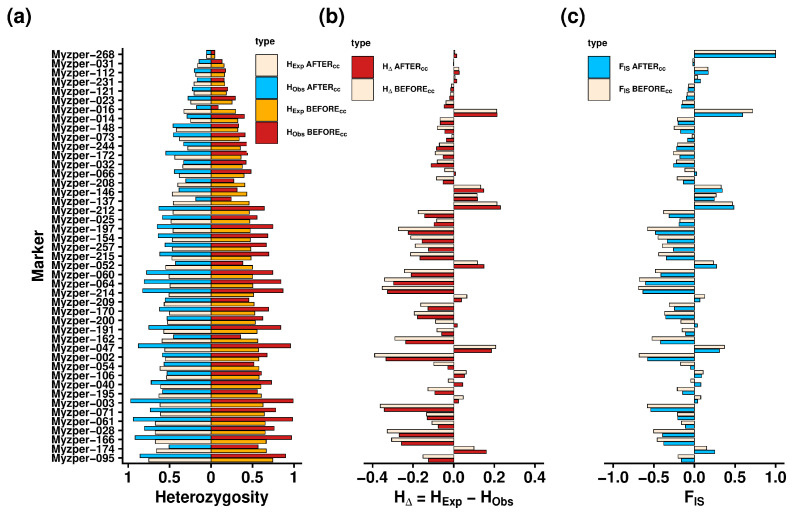
Distribution of the selected population descriptive parameters across SSR loci before (B_cc_) and after (A_cc_) clone correction: (**a**) observed heterozygosity (H_Obs_) and expected heterozygosity (H_Exp_); (**b**) heterozygosity difference (HΔ); and (**c**) inbreeding coefficient (F_IS_). Please note the different scales on the x axis.

Exploratory data analysis revealed one phylogenetically uninformative marker (Myzper-207) with a minor allele frequency (MAF) below the predefined threshold of 0.01. Therefore, this locus was excluded from the population analysis. A total of 180 alleles were identified (194 when all 49 markers were counted), with a mean and median of 4 alleles per locus and a range of 2 and 8 alleles per locus (Table 2). Before clone correction (B_cc_) was performed, the observed heterozygosity (H_Obs_) ranged between 0.045 and 0.989 (mean = 0.547, median = 0.559), while the expected heterozygosity (H_Exp_) ranged between 0.044 and 0.745 (mean = 0.46, median = 0.482), and the inbreeding coefficient (F_IS_) ranged between −0.69 and 0.714 (mean = −0.157, median = −0.187). Within the filtered dataset (N = 365), 97 distinct multilocus genotypes (MLGs) were identified, and only one representative per MLG was retained and re-evaluated after clone correction (A_cc_). After clone correction was performed, the values of H_Obs_ ranged between 0.056 and 0.969 (mean = 0.533, median = 0.553), the values of H_Exp_ ranged between 0.054 and 0.75 (mean = 0.466, median = 0.482), and the values of F_IS_ ranged between −0.63 and 0.597 (mean = −0.121, median = −0.149). Many loci showed heterozygote excess in both cases (B_cc_ and A_cc_), which aligned with the negative values of the inbreeding coefficient (Figure 4).

The analysis also aimed to test for HWE on the global (whole genome) and local (locus specific) scales. Both the paired Welch’s test (B_cc_: *p* = 0.999, A_cc_: *p* = 0.998) and Wilcoxon signed-rank test (B_cc_: *p* = 0.999, A_cc_: *p* = 0.999) did not show statistically significant differences in HWE on the global scale. Non-parametric Wilcoxon signed-rank test was applied as a supplementary test when the exploratory data analysis indicated potential violation of parametric model assumptions for some of the tested variants (Table S3, Figure S2). In contrast, a substantial number of loci (31–41) showed statistically significant deviation from HWE when tested with the $\chi^{2}$-test and exact test (Table S4).

The population was further characterised by employing diversity indices, where both the Shannon–Weaver index (B_cc_: H = 3.06; A_cc_: H = 4.57) and Stoddard and Taylor’s index (B_cc_: G = 7.85; A_cc_: G = 97) suggested high diversity, whereas the values of the Simpson’s index (B_cc_: λ = 0.873; A_cc_: λ = 0.99) and evenness (B_cc_: E_5_ = 0.337; A_cc_: E_5_ = 1) led to the opposite conclusion. The standardized index of association (B_cc_: ${\overset{´}{r}}_{D}$ = 0.365, *p* = 0.001; A_cc_: ${\overset{´}{r}}_{D}$ = 0.221, *p* = 0.001) showed moderate-to-high LD, though the paired association (paired-${\overset{´}{r}}_{D}$) index revealed several pairs of loci (B_cc_: 85 out of 990 combinations, i.e., ~8.48% pairs; A_cc_: 196 out of 990 combinations, i.e., ~19.8% pairs) with very low or no LD (Figures S3 and S4).

**Figure 5:**
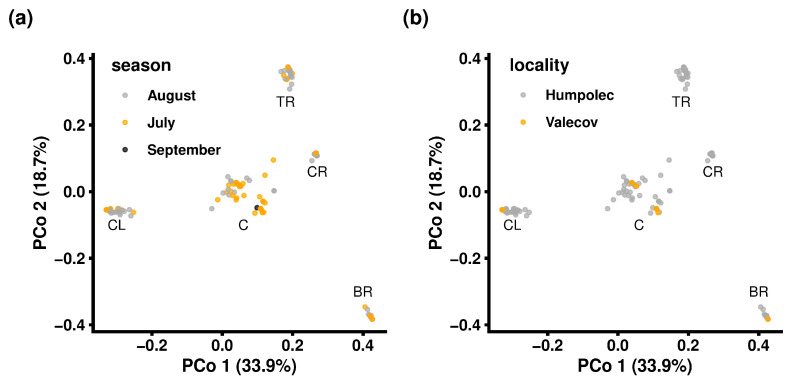
Population stratified by season (**a**) and locality (**b**), where shortcuts refer to the approximate position of the clusters identified by PCoA. BR = bottom right, C = centre, CL = centre left, CR = centre right, TR = top right.

**Figure 6:**
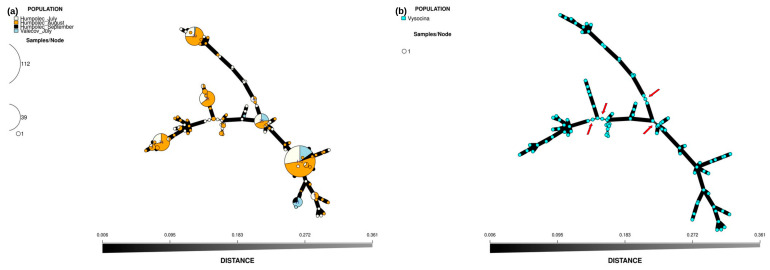
Population structure and genetic distances based on MSN analysis, including spatiotemporal information: before clone correction (**a**) and after clone correction (**b**). Red arrows highlight where distant lineages begin.

Exploratory analysis via PCoA led to the identification of five distinct clusters (Figure 5), with no apparent association with seasonality (Figure 5a) or locality (Figure 5b). Similar results were obtained by the minimum spanning network (MSN), showing the connection and genetic distances between aphid accessions (Figure 6). Five main lineages were identified (Figure 6b), and each lineage included at least one of the highly abundant MLGs, namely MLG.58 (112 clones; 78.32% share in the “CL” cluster), MLG.16 (39 clones; 81.25% share in the “BR” cluster), MLG.36 (34 clones; 56.67% share in the “TR” cluster), MLG.11 (28 clones; 75.68% share in the “CR” cluster) and MLG.82 (25 clones; 32.47% share in the “C” cluster). For a deeper understanding of the underlying structure, population descriptive parameters were estimated for each identified cluster in the same manner as previously described for the higher hierarchical level represented by all accessions identified in the Vysočina region (Tables S5–S8, Figures S5 and S6).

## 4. Discussion

### 4.1. In Silico Analysis and Development of SSR Markers

The initial analytical step was a comparison of SSR distribution and abundancy with that of other insect species. The number of pure SSRs is 2–3 times higher than the median value obtained across 29 beetle species [55], yet it is at least 10 times lower compared with *Tapinoma indicum* [56]. These substantial differences are probably due to the differences in genome size, as larger genomes tend to have more microsatellite loci, and the evolutionary history of the given species [57]. Dinucleotide microsatellites with AT-rich motifs represent the most abundant type (~2/3 of all SSRs), which is in accordance with the pattern observed in other insect species [57].

The high abundance of dinucleotide microsatellites provides partial explanation as to why the initial number of candidate loci was relatively low. The primary objective of the study was to develop reliable SSR markers, and according to our experience, dinucleotide microsatellites are more error-prone than any other SSRs. This is due to the elevated level of stutter ratio, which could lead to difficulties in resolving a true heterozygote from a homozygote with a stutter band [58,59]. The signal-to-noise ratio is typically improved with a longer motif, though other factors, including the number of repetitions or motif composition, might also be important [60]. Therefore, all dinucleotide SSRs were excluded herein and trinucleotide microsatellites, as the second most abundant type, were targeted instead.

The number of candidate loci was further reduced with the application of additional criteria, including a single-copy locus per genome, a minimal number of repeats, a clean profile on agarose and capillary electrophoresis, or locus specificity as verified by sequencing. Interestingly, the sequencing profile of several loci suggested a mixture of DNA stretches with different numbers of repeats, resulting in ambiguous sequence signal (Figure S7). This phenomenon may be attributed to the sequencing of heterozygous genotypes with different numbers of microsatellite repetitions per allele, but we obtained the same results even for bands cut out of polyacrylamide gel (unpublished results). Another explanation could be chromosomal rearrangements causing the duplication of some loci [4,61], but answering this question would require additional research. For the sake of clarity, we decided to omit these loci as we could not fully characterise them.

The last step involved multiplexing the markers to conduct high-throughput screening of hundreds or even thousands of samples, allowing for increased speed and reduced costs [62]. The high level of multiplexing renders the developed SSR markers competitive with massive parallel sequencing (MPS), which is costly for field studies with large numbers of samples and requires advanced bioinformatic skills. Considering the utilisation of MPS in analyses of larger numbers of aphid samples, an estimation was made to compare with the price of MPS for a typical set of 94 samples, which is commonly offered by local commercial providers (Figure S8). The figure compares the expected costs of whole-genome sequencing (WGS) with the costs of using our SSR panel for comprehensive analysis. The costs of WGS for a 382 Mb aphid genome were ~10× coverage (approx. 375 Gb of raw data) with only basic bioinformatic service (i.e., demultiplexing and adaptor removal). The figure demonstrates that the costs per sample when using the SSR analysis are still lower. However, it is possible to apply a compromised approach, where amplification of multiplexed SSRs allows for the targeting of reliable polymorphic loci while reducing the genome complexity for genotyping using MPS [63,64]. Therefore, we believe that our SSR markers would be useful even in the genomic era.

### 4.2. Internal Validation

Genotyping error is an inevitable component of any research study, and undetected error can significantly bias the interpretation of results. Such error can arise at all stages of the research process, as there are numerous sources of error and no simple solution to the problem. Nonetheless, in conjunction with other precautions, the impact of error can be minimized using well-characterised and highly reproducible markers. Therefore, the genotyping error of the SSR markers identified in this study was estimated on several levels. The majority of the SSR markers (40 out of 49) exhibited a desirable 0% mean error rate per allele and locus. Conversely, several loci (3 out of 49) were identified as highly error-prone. Fortunately, removing these loci from the dataset lowered all estimated error rates to an acceptable level comparable with other studies [65,66,67]. Of course, when lower error rates are crucial—for example, in the case of parentage analysis—additional error-prone loci like Myzper-052 and Myzper-268 could be excluded; however, these loci were retained in this study as a compromise between increased informational value and increased data bias. Surprisingly, no published studies dealing with the genotyping error of heavily exploited SSR markers for *M. persicae* [21,23] were found, and thus, any direct comparison could not be made. The only investigation of the quality of several commonly used SSR markers (Myz9, M35, M40, M49, M63 and M86) was provided by Wouters in his doctoral thesis, mentioning the occurrence of unspecific amplicons and a high inaccuracy of all tested markers [68].

The species specificity of the *M. persicae* SSR markers was tested across several higher-order taxa of both animal and plant species, where all SSR markers failed to amplify and many markers seemed to be transferable to *P. humuli* species. However, limited transferability was observed for the more evolutionary distant species *A. nasturtii* and *A. fabae*. Transferability of SSR markers between related species is a common phenomenon [24] and could be perceived in both a positive and negative way with regard to purpose. Markers developed for one species may be utilised for studies in other species with limited genetic information, but they could also bring noise into the data (e.g., higher occurrence of null alleles). In our case, the SSR assays facilitated the rapid discrimination between the morphologically similar species *M. persicae* and *P. humuli*. Any *P. humuli* samples were automatically discarded during the filtering process due to the high portion of non-amplifying loci. Nevertheless, these transferable markers can be subsequently tested for use in studies of the population dynamics of *P. humuli*.

### 4.3. Population Analysis

The PCoA and MSN revealed the presence of several genetic clusters with no apparent association with geographical localities or seasonal dynamics. This raised the question of whether the analysed accessions should be treated as belonging to one or more populations. The correct deciphering of population structure is a key aspect for most population genetic studies, as many population parameters and statistical methods (e.g., AMOVA) rely on a predefined number of genetic groups. At first sight, several clues indicated five populations. The strong-to-moderate linkage disequilibrium (LD) signal was diminished in most clusters (Table S7) after clone correction, and the F_ST_ values suggested strong differentiation among groups (Table S8). On the other hand, additional evidence supported the interpretation of a single panmictic population. The differences between the H_Exp_ and H_Obs_ ($H_{\Delta }$) and F_IS_ values were less extreme (Table S5) when compared with the genetic clusters, and only one MLG was found to dominate within each cluster. This dominance of one single MLG aligns with the occurrence of many monomorphic loci (Figure S5) and the polarized H_Obs_ values (0 or 1) for most loci (Figure S6).

Further research including more geographically distant localities and sampling aphids across multiple seasons would provide a clearer answer regarding the aphid population structure and dynamics in Czechia. Currently, the aphid accessions are considered as belonging to a single population composed of five prevailing aphid lineages at the deeper population level, as this conclusion provides a more plausible biological interpretation. The samples consisted of parthenogenetically derived migrants, and the observed clusters are therefore connected to the respective fundatrices of the original fundatrigeniae. Therefore, it can be assumed that the current population arose from 5 to 97 fundatrices, as 97 MLGs were identified, and the detected pattern reflects the genetic distances between them. Of course, this estimation is approximate and may be subjected to potential biases due to genotyping error [66], new mutations [17,69] or recombination—although this has only been observed in ants [70] and bees [71] and its occurrence in aphids remains a topic of debate [72,73].

The studied aphid population showed the typical characteristics expected of a species with clonal or partially clonal reproduction [74]. The proportion of multilocus genotypes (MLGs) to the total number of samples was significantly below 1 (~0.27), negative F_IS_ values were detected across most loci, and moderate-to-strong LD values were found. The obtained LD values (B_cc_: 0.37; A_cc_: 0.22) are consistent with the idea of cyclic parthenogenesis, where the LD values for fully asexual populations should be equal to one, while the values should be close or equal to zero for sexually reproducing populations, in cases where no other evolutionary forces like selection or genetic drift are involved. The sexual mode of reproduction, which is typical for autumn generations of aphids, leads to, at least partially, the interruption of linkage between some pairs of loci. The same phenomenon is probably responsible for restoring the Hardy–Weinberg equilibrium in several SSR loci (B_cc_: 4 loci, A_cc_: 14 loci). These findings are in agreement with the diversity index values, although the Shannon–Weaver and Stoddard and Taylor’s indices provide seemingly contradictory results when compared with the Simpson’s index and evenness indicator. Nonetheless, these indices may be strongly influenced by the ratio of diversity to evenness and by the population size [75,76].

## 5. Conclusions

A new set of 49 comprehensively characterised SSR markers was developed, most of which exhibit many desirable characteristics, including single-locus specificity, known chromosome position, moderate-to-high polymorphism and low genotyping error rates. The developed markers are amenable to multiplexing and their organization into four multiplex assays enables cheaper and faster analysis when compared with singleplex approaches. Furthermore, the population study presented herein demonstrates their high discriminatory power, which is essential for elucidating the population structure and dynamics of *M. persicae*.

## Figures and Tables

**Table 1:** Overview of the developed SSR markers.

Assay	Marker	c [μM]	Dye	Primer (5′-3′)
1	Myzper-023	0.1	VIC	F: ACTAATTGCCGAATGTGCG
				R: GGTCGTCCAGTTCATTCTTACT
1	Myzper-064	0.2	6-FAM	F: CAGATGGTGCTCGTTCCCTA
				R: GCTTGAGACAGAACCCGACA
1	Myzper-095	0.1	VIC	F: ACAGCGTATCGTCGTTTTGG
				R: TGTTGATTGTTGTTCCCCACT
1	Myzper-112	0.3	PET	F: TTTCGGACAAAAGGGTTTCATAC
				R: CAGCTCCGTCCATCCATC
1	Myzper-121	0.2	VIC	F: GATTTGATCCCTGCAATTTCCC
				R: CCGTCGCCCAGTCTTGAT
1	Myzper-131	0.1	6-FAM	F: AGCAATAAGTGGAAACCGTCG
				R: CCTTTTACCTGCGTCGTCC
1	Myzper-137	0.2	6-FAM	F: CCCGCAGTTTAAATCCGTG
				R: CAACCCCGCAAAATCCAT
1	Myzper-146	0.2	NED	F: GGCCTGCTATATATACGGACCA
				R: CATGCAACCACCGTCGTAG
1	Myzper-180	0.2	6-FAM	F: CGCTCTACCCCTTTGAATGG
				R: GGCAGTTCATAGACCTTCGGAC
1	Myzper-207	0.1	6-FAM	F: GGTATTTCTGCCATAGGAGGAGC
				R: CTGTCCCAAACCAACCACTG
1	Myzper-209	0.2	NED	F: GTACAATGACTGACAAGCAAAACA
				R: AAATCCCAAAGCCTCACGTC
1	Myzper-231	0.3	PET	F: ACCTCCCCTGTACTTACCTTCA
				R: TGAAAGTATTGAATGATCCGGCT
2	Myzper-016	0.1	6-FAM	F: CATTTTGCAGACAGACGCC
				R: AGGTATATAATGTCCGCTGAGAAGA
2	Myzper-025	0.3	PET	F: GTCCAACCCTAACCGCTACT
				R: GGGTGTTGTATGCTGGGAAC
2	Myzper-054	0.1	6-FAM	F: TAGAAGTCCCACCGCTGTTT
				R: GCATAGATGGTGTTGGAGGT
2	Myzper-060	0.2	NED	F: AGCTACCCTCCTTTTCGGTAC
				R: GATGTCGCTCGCTCACAAG
2	Myzper-061	0.1	6-FAM	F: GCAGCCAAAGTTTCGTTTACC
				R: ACGCCTTGGGTTGATTTTACA
2	Myzper-066	0.1	VIC	F: CGGCTTTGATGTCGTTAATGAG
				R: CATGTTACACGCACGCTGG
2	Myzper-073	0.1	6-FAM	F: CATTGATCATGCTGACTGGC
				R: ATACCTACTGACACGCACGG
2	Myzper-172	0.2	NED	F: TTATCATTGTCATCGGCAGAAAC
				R: TGCACCTCCCCACATTACTC
2	Myzper-174	0.2	NED	F: GCCGAGCAAATAATTAACGAAG
				R: GAAGACTTTTGACAACCGCC
2	Myzper-182	0.1	VIC	F: GTCGCACCCACTGTTATTACCA
				R: TGGATGTGACGGCAACAGT
2	Myzper-197	0.3	PET	F: GTAACTTATTCAATAATCCCCTCGG
				R: TGTCACTTCAACACGAACCA
2	Myzper-208	0.3	6-FAM	F: GGATATGAAGGGGAATCAGCC
				R: GTCGAGGCTTCCACCATAATAA
2	Myzper-212	0.3	PET	F: AATTTGTAACTCACGTCCACTCG
				R: AACAACCATATTCCGCAGACC
3	Myzper-028	0.1	6-FAM	F: CACTCACGTTTAGTTGCGGG
				R: TGCTTCTTTGCTTGCTGGAA
3	Myzper-040	0.2	NED	F: GGATGTGGGTGTGTACGAGTA
				R: CCGGGATAGAGTAAGGCAAAG
3	Myzper-047	0.2	PET	F: TGACCCACACACGGAATTTAT
				R: CATACACCTTTGGCGGAATC
3	Myzper-106	0.2	6-FAM	F: TTCGATTCCACTGTTGCTGC
				R: TGTACTTTGCGGGTTTTCGG
3	Myzper-148	0.3	PET	F: TTCCTGGTTGTTGGTATACGAA
				R: TGAAATATAGCTCACGACGATGT
3	Myzper-166	0.1	VIC	F: GACCTCGGCTTTATGAATATCAAA
				R: CGAGATGCTGTGTTAAACGC
3	Myzper-191	0.2	6-FAM	F: CGGGGCTTAGTACCTTCGAA
				R: GGAGATGCGTACCTTTGCG
3	Myzper-200	0.2	VIC	F: TTCGGACGTTCTATGGGTG
				R: CTTTACATCATCGCCACCG
3	Myzper-214	0.2	NED	F: GCTGACAATCCCCAACGAC
				R: CGAAAGGGCGAATTTGATGC
3	Myzper-215	0.2	VIC	F: GAACGGTGTCAGAGGAGTGTC
				R: CGATGTATTGGCTGTAGACGAC
3	Myzper-244	0.1	6-FAM	F: CGGTGCGTGCAATAATATCC
				R: CAACGGGCATTGAGGAGAA
3	Myzper-257	0.1	6-FAM	F: ATGTGGATGATTGCGGTGT
				R: ACCGAACGTATTGATTAGACCC
4	Myzper-002	0.1	6-FAM	F: GGTCCTCTATTAGCCTCCGC
				R: ATAGAGCAACAGCAGCACCG
4	Myzper-003	0.3	PET	F: GTATACCTGCGCACCGAGTT
				R: AGTCATGGTCAGCACAGTCG
4	Myzper-014	0.1	NED	F: TCCGAGGGTAGGTAGGTATATCC
				R: CCTGCTCCACTTCCCCTA
4	Myzper-031	0.1	VIC	F: ATGCAGCGACCTCCAAATG
				R: TTATTGTTCTGCTCGCACGG
4	Myzper-032	0.2	NED	F: ATCCACCCACGCAACAAC
				R: CAGAAAACGAAAGGGGACG
4	Myzper-052	0.1	VIC	F: GCAGCGATCAACATCAAACG
				R: CCACTGTCCCGAATTGTAGC
4	Myzper-071	0.2	6-FAM	F: GAAAACCACTAGCACTCGACG
				R: TGTCTTTGGTGGTGTCCTCA
4	Myzper-154	0.2	6-FAM	F: GCTCACACCGTAACGCAC
				R: CGCGACTATGACGACGTTG
4	Myzper-162	0.1	6-FAM	F: GGTTAGGTTGCTCGTTGGAATC
				R: CTTTGCGACTCACTTCCGAC
4	Myzper-170	0.3	PET	F: ATAGCGTGAGGGTTTGGGA
				R: TGCCTGGTCAGCGAAATATT
4	Myzper-195	0.2	NED	F: CGCCGCCAAGCTGTAGTA
				R: GCCGACAATGCCAATAAAGATAA
4	Myzper-268	0.2	VIC	F: GGCCTGCGTACCGTGTTG
				R: CGTACACCCGACAACCCG

**Table 2:** Genetic characterisation of the studied loci.

Assay	Marker	N_a_ ^a^	Chr ^b^	Hit From ^c^	Hit to ^c^	Strand	Motif	Repeats
1	Myzper-023	3	chr 1	23,347,209	23,347,245	Plus	TAT	12
1	Myzper-064	3	chr 4	4,678,265	4,678,295	Plus	ATT	10
1	Myzper-095	7	chr 2	59,306,409	59,306,454	Plus	CAA	15
1	Myzper-112	3	chr 6	18,695,671	18,695,695	Plus	AAT	8
1	Myzper-121	4	chr 2	17,196,081	17,196,051	Minus	ACA	10
1	Myzper-131	4	chr 6	9,082,535	9,082,556	Plus	CGG	7
1	Myzper-137	3	chr 2	6,492,290	6,492,314	Plus	AAT	8
1	Myzper-146	3	chr 2	31,378,493	31,378,526	Plus	TAA	11
1	Myzper-180	4	chr 6	23,237,826	23,237,802	Minus	CGC	8
1	Myzper-207	2	chr 1	10,650,144	10,650,165	Plus	GGA	7
1	Myzper-209	6	chr 4	21,197,304	21,197,328	Plus	ATG	8
1	Myzper-231	3	chr 2	81,831,810	81,831,765	Minus	ACCTA	9
2	Myzper-016	3	chr 4	6,305,555	6,305,531	Minus	GTC	8
2	Myzper-025	4	chr 3	51,486,311	51,486,347	Plus	TAA	12
2	Myzper-054	4	chr 6	6,395,254	6,395,221	Minus	TTG	11
2	Myzper-060	3	chr 4	20,391,192	20,391,216	Plus	ATT	8
2	Myzper-061	6	chr 3	39,954,022	39,954,050	Plus	GTGC	7
2	Myzper-066	4	chr 2	17,970,961	17,970,991	Plus	AAT	10
2	Myzper-073	3	chr 3	47,724,216	47,724,186	Minus	ATT	10
2	Myzper-172	4	chr 4	6,063,778	6,063,733	Minus	ATT	15
2	Myzper-174	4	chr 2	2,303,608	2,303,638	Plus	ATT	10
2	Myzper-182	4	chr 5	28,211,033	28,211,063	Plus	CCG	10
2	Myzper-197	2	chr 3	40,651,902	40,651,875	Minus	TTA	9
2	Myzper-208	3	chr 1	70,791,470	70,791,451	Minus	TTA	10
2	Myzper-212	3	chr 6	12,847,813	12,847,789	Minus	TAT	8
3	Myzper-028	8	chr 4	26,556,362	26,556,326	Minus	TGC	12
3	Myzper-040	6	chr 6	1,510,305	1,510,338	Plus	CTG	11
3	Myzper-047	4	chr 1	11,044,892	11,044,922	Plus	ATA	10
3	Myzper-106	3	chr 1	36,719,415	36,719,394	Minus	CCG	7
3	Myzper-148	3	chr 1	18,060,929	18,060,953	Plus	TCG	8
3	Myzper-166	5	chr 1	31,830,661	31,830,691	Plus	ATT	10
3	Myzper-191	4	chr 4	2,355,865	2,355,889	Plus	CAG	8
3	Myzper-200	3	chr 3	34,124,313	34,124,280	Minus	ATT	11
3	Myzper-214	3	chr 1	33,520,102	33,520,123	Plus	GAC	7
3	Myzper-215	4	chr 2	30,879,299	30,879,248	Minus	CAA	17
3	Myzper-244	3	chr 4	58,683,634	58,683,604	Minus	TAT	10
3	Myzper-257	2	chr 6	6,832,400	6,832,376	Minus	TCG	8
4	Myzper-002	6	chr 4	57,017,450	57,017,471	Plus	CGC	7
4	Myzper-003	7	chr 6	10,043,860	10,043,809	Minus	TAA	17
4	Myzper-014	2	chr 5	286,153	286,177	Plus	ATT	8
4	Myzper-031	4	chr 2	51,632,413	51,632,389	Minus	TCG	8
4	Myzper-032	2	chr 6	17,071,054	17,071,075	Plus	CGT	7
4	Myzper-052	5	chr 1	75,106,271	75,106,244	Minus	GCG	9
4	Myzper-071	5	chr 2	18,003,582	18,003,558	Minus	TCC	8
4	Myzper-154	2	chr 1	2,532,569	2,532,596	Plus	TAT	9
4	Myzper-162	6	chr 5	22,618,169	22,618,148	Minus	CCG	7
4	Myzper-170	6	chr 4	17,291,633	17,291,612	Minus	ATA	7
4	Myzper-195	6	chr 2	22,424,405	22,424,429	Plus	CTG	8
4	Myzper-268	3	chr 4	65,308,976	65,308,952	Minus	GAC	8

Note: ^a^ number of alleles, ^b^ chromosome number, ^c^ BLAST hits against aphid genome (GCA_029232275.1) assembled on the chromosomal level. The exact position of the microsatellite loci might be slightly shifted (by several bp) due to differences between the aligned sequence and the assembled genome in the flanking regions.

## Data Availability

The sequencing data were submitted to GenBank under the following identification numbers: PX116999-PX117001 and PX148051-PX148096. The raw data supporting the conclusions of this article will be made available by the authors upon request.

## Supplementary Materials

The following supporting information can be downloaded at: https://www.mdpi.com/article/10.3390/insects16111156/s1, Table S1: Mean error rate per allele (*e_a_*) and per locus (*e_l_*); Table S2: SSRs transferability (+) between species; Table S3: Normality and homoskedasticity check for expected heterozygosity (H_Exp_) and observed heterozygosity (H_Obs_). Statistically significant values are highlighted; Table S4: *p*-values of HWE. Statistically significant values are highlighted; Table S5: Population descriptors for each PCoA cluster before and after clone correction part 1—observed heterozygosity (H_Obs_), expected heterozygosity (H_Exp_), inbreeding coefficient (F_IS_); Table S6: Population descriptors for each PCoA cluster before and after clone correction part 2—Shannon-Weaver index (H), Taylor’s index (G), Simpson’s diversity index (*λ*); Table S7: Population descriptors for each PCoA cluster before and after clone correction part 3—evenness (E) and standardized association index ($\bar{r}$*D*); Table S8: Pairwise F_ST_ values before (blue) and after (red) clone correction; Figure S1: Initial allelic ladder. Multiplex assay 1–4 (a–d); Figure S2: Exploratory data analysis of expected heterozygosity (H_Exp_) before (a) and after (c), clone correction and observed heterozygosity (H_Obs_) before (b) and after (d) clone correction; Figure S3: Paired $\bar{r}$*D* before clone correction. Depicted values are *p*-values for given pair of loci. Color heatmap reflects degree of association between loci; Figure S4: Paired $\bar{r}$*D* after clone correction. Depicted values are *p*-values for given pair of loci. Color heatmap reflects degree of association between loci; Figure S5: Number of alleles per locus per PCoA cluster. Shortcuts refer to approximate position of identified clusters by PCoA. BR = bottom right, C = center, CL = center left, CR = center right, TR = top right; Figure S6: Observed (H_Obs_) and expected (H_Exp_) heterozygosity per cluster identified by PCoA. Shortcuts refer to approximate position of identified clusters by PCoA. BR = bottom right, C = center, CL = center left, CR = center right, TR = top right; Figure S7: Example of Sanger sequencing with ambiguous sequence signal; Figure S8: Estimated price of whole genome sequencing (WGS) and SSR analysis. Rough estimation of costs per common sample set (1 set = 94 samples). Initial parameters for WGS as a service are ~10× coverage, approx. 375 Gb of raw data, without bioinformatic service. Price per library and sequencing costs were adopted from pricelist of local sequencing service provider.

## References

[1] Barbagallo S., Cocuzza G., Cravedi P., Komazaki S. (2007). IPM Case Studies: Deciduous Fruit Trees. Aphids as Crop Pests.

[2] Field L.M., Blackman R.L., Tyler-Smith C., Devonshire A.L. (1999). Relationship Between Amount of Esterase and Gene Copy Number in Insecticide-Resistant *Myzus Persicae* (Sulzer). Biochem. J..

[3] Manicardi G.C., Nardelli A., Mandrioli M. (2015). Fast Chromosomal Evolution and Karyotype Instability: Recurrent Chromosomal Rearrangements in the Peach Potato Aphid *Myzus persicae* (Hemiptera: Aphididae). Biol. J. Linn. Soc..

[4] Mathers T.C., Wouters R.H.M., Mugford S.T., Swarbreck D., van Oosterhout C., Hogenhout S.A. (2020). Chromosome-Scale Genome Assemblies of Aphids Reveal Extensively Rearranged Autosomes and Long-Term Conservation of the X Chromosome. Mol. Biol. Evol..

[5] Blackman R.L. (1980). Chromosome Numbers in the Aphididae and Their Taxonomic Significance. Syst. Entomol..

[6] Blackman R.L., Takada H., Kawakami K. (1978). Chromosomal Rearrangement Involved in Insecticide Resistance of *Myzus persicae*. Nature.

[7] Li Y., Zhang B., Moran N.A. (2020). The Aphid X Chromosome Is a Dangerous Place for Functionally Important Genes: Diverse Evolution of Hemipteran Genomes Based on Chromosome-Level Assemblies. Mol. Biol. Evol..

[8] Jaquiéry J., Rispe C., Roze D., Legeai F., Le Trionnaire G., Stoeckel S., Mieuzet L., Da Silva C., Poulain J., Prunier-Leterme N. (2013). Masculinization of the X Chromosome in the Pea Aphid. PLoS Genet..

[9] Blackman R.L. (1974). Life-Cycle Variation of *Myzus persicae* (Sulz.) (Hom., Aphididae) in Different Parts of the World, in Relation to Genotype and Environment. Bull. Entomol. Res..

[10] Blackman R.L. (1987). Reproduction, Cytogenetics and Development. Aphids Their Biol. Nat. Enemies Control.

[11] Blackman R., Eastop V. (2000). Aphids World’s Crops: An Identification and Information Guide.

[12] Vorburger C., Lancaster M., Sunnucks P. (2003). Environmentally Related Patterns of Reproductive Modes in the Aphid *Myzus persicae* and the Predominance of Two ‘Superclones’ in Victoria, Australia. Mol. Ecol..

[13] Poupoulidou D., Margaritopoulos J.T., Kephalogianni T.E., Zarpas K.D., Tsitsipis J.A. (2006). Effect of Temperature and Photoperiod on the Life Cycle in Lineages of *Myzus persicae Nicotianae* and *Myzus persicae* s. Str. (Hemiptera: Aphididae). Eur. J. Entomol..

[14] Yan S., Wang W., Shen J. (2020). Reproductive Polyphenism and Its Advantages in Aphids: Switching Between Sexual and Asexual Reproduction. J. Integr. Agric..

[15] Roy S.W. (2021). Inbreeding, Male Viability, and the Remarkable Evolutionary Stability of the Aphid X Chromosome. Heredity.

[16] Delmotte F., Leterme N., Gauthier J.-P., Rispe C., Simon J.-C. (2002). Genetic Architecture of Sexual and Asexual Populations of the Aphid *Rhopalosiphum padi* Based on Allozyme and Microsatellite Markers. Mol. Ecol..

[17] Kanbe T., Akimoto S. (2009). Allelic and Genotypic Diversity in Long-Term Asexual Populations of the Pea Aphid, *Acyrthosiphon pisum* in Comparison with Sexual Populations. Mol. Ecol..

[18] Li J., Cao J., Niu J., Liu X., Zhang Q. (2015). Identification of the Population Structure of *Myzus persicae* (Hemiptera: Aphididae) on Peach Trees in China Using Microsatellites. J. Insect Sci..

[19] Mathers T.C., Mugford S.T., Percival-Alwyn L., Chen Y., Kaithakottil G., Swarbreck D., Hogenhout S.A., Oosterhout C. (2019). van Sex-Specific Changes in the Aphid DNA Methylation Landscape. Mol. Ecol..

[20] Jarne P., Lagoda P.J.L. (1996). Microsatellites, from Molecules to Populations and Back. Trends Ecol. Evol..

[21] Sloane M.A., Sunnucks P., Wilson A.C.C., Hales D.F. (2001). Microsatellite Isolation, Linkage Group Identification and Determination of Recombination Frequency in the Peach-Potato Aphid, *Myzus persicae* (Sulzer) (Hemiptera: Aphididae). Genet. Res..

[22] Wilson A.C.C., Sunnucks P., Blackman R.L., Hales D.F. (2002). Microsatellite Variation in Cyclically Parthenogenetic Populations of *Myzus persicae* in South-Eastern Australia. Heredity.

[23] Wilson A.C.C., Massonnet B., Simon J.-C., Prunier-Leterme N., Dolatti L., Llewellyn K.S., Figueroa C.C., Ramirez C.C., Blackman R.L., Estoup A. (2004). Cross-Species Amplification of Microsatellite Loci in Aphids: Assessment and Application. Mol. Ecol. Notes.

[24] Weng Y., Azhaguvel P., Michels G.J., Rudd J.C. (2007). Cross-Species Transferability of Microsatellite Markers from Six Aphid (Hemiptera: Aphididae) Species and Their Use for Evaluating Biotypic Diversity in Two Cereal Aphids. Insect Mol. Biol..

[25] Feng H., Chen W., Hussain S., Shakir S., Tzin V., Adegbayi F., Ugine T., Fei Z., Jander G. (2023). Horizontally Transferred Genes as RNA Interference Targets for Aphid and Whitefly Control. Plant Biotechnol. J..

[26] Mathers T.C., Chen Y., Kaithakottil G., Legeai F., Mugford S.T., Baa-Puyoulet P., Bretaudeau A., Clavijo B., Colella S., Collin O. (2017). Rapid Transcriptional Plasticity of Duplicated Gene Clusters Enables a Clonally Reproducing Aphid to Colonise Diverse Plant Species. Genome Biol..

[27] Wang X., Wang L. (2016). GMATA: An Integrated Software Package for Genome-Scale SSR Mining, Marker Development and Viewing. Front. Plant Sci..

[28] R Core Team (2024). R: A Language and Environment for Statistical Computing. https://www.r-project.org/.

[29] Wickham H., François R., Henry L., Müller K., Vaughan D. (2023). Dplyr: A Grammar of Data Manipulation. https://cran.r-project.org/web/packages/dplyr/index.html.

[30] Wickham H., Vaughan D., Girlich M. (2024). Tidyr: Tidy Messy Data. https://cran.r-project.org/web/packages/tidyr/index.html.

[31] Wickham H., Henry L. (2023). Purrr: Functional Programming Tools. https://cran.r-project.org/web/packages/purrr/index.html.

[32] Ooms J. (2014). The Jsonlite Package: A Practical and Consistent Mapping Between JSON Data and R Objects. arXiv.

[33] Wickham H. (2023). Stringr: Simple, Consistent Wrappers for Common String Operations. https://cran.r-project.org/web/packages/stringr/index.html.

[34] Wickham H. (2016). Ggplot2: Elegant Graphics for Data Analysis. https://ggplot2.tidyverse.org/.

[35] Pedersen T.L. (2024). Patchwork: The Composer of Plots. https://cran.r-project.org/web/packages/patchwork/index.html.

[36] Slowikowski K. (2024). Ggrepel: Automatically Position Non-Overlapping Text Labels with ‘Ggplot2’. https://cran.r-project.org/web/packages/ggrepel/index.html.

[37] Gohel D., Skintzos P. (2025). Flextable: Functions for Tabular Reporting. https://cran.r-project.org/web/packages/flextable/index.html.

[38] Xie Y. (2025). Knitr: A General-Purpose Package for Dynamic Report Generation in R. https://yihui.org/knitr/.

[39] Allaire J., Xie Y., Dervieux C., McPherson J., Luraschi J., Ushey K., Atkins A., Wickham H., Cheng J., Chang W. (2024). Rmarkdown: Dynamic Documents for R.

[40] Vašek J., Čílová D., Melounová M., Svoboda P., Zdeňková K., Čermáková E., Ovesná J. (2021). OpiumPlex Is a Novel Microsatellite System for Profiling Opium Poppy (*Papaver somniferum* L.). Sci. Rep..

[41] Zhang Z., Schwartz S., Wagner L., Miller W. (2000). A Greedy Algorithm for Aligning DNA Sequences. J. Comput. Biol..

[42] Untergasser A., Cutcutache I., Koressaar T., Ye J., Faircloth B.C., Remm M., Rozen S.G. (2012). Primer3new Capabilities and Interfaces. Nucleic Acids Res..

[43] (2014). Aphids on Potato, Sugar Beet, Pea, Broad Bean and Other Vegetables.

[44] Hall T. (1996). BioEdit: A User-Friendly Biological Sequence Alignment Editor and Analysis Program for Windows 95/98/NT. Nucleic Acids Symp. Ser..

[45] Madeira F., Madhusoodanan N., Lee J., Eusebi A., Niewielska A., Tivey A.R.N., Lopez R., Butcher S. (2024). The EMBL-EBI Job Dispatcher Sequence Analysis Tools Framework in 2024. Nucleic Acids Res..

[46] Brown S.S., Chen Y.-W., Wang M., Clipson A., Ochoa E., Du M.-Q. (2017). PrimerPooler: Automated Primer Pooling to Prepare Library for Targeted Sequencing. Biol. Methods Protoc..

[47] Pompanon F., Bonin A., Bellemain E., Taberlet P. (2005). Genotyping Errors: Causes, Consequences and Solutions. Nat. Rev. Genet..

[48] Jombart T. (2008). Adegenet: A r Package for the Multivariate Analysis of Genetic Markers. Bioinformatics.

[49] Paradis E. (2010). Pegas: An r Package for Population Genetics with an Integrated–Modular Approach. Bioinformatics.

[50] Goudet J., Jombart T. (2022). Hierfstat: Estimation and Tests of Hierarchical f-Statistics. https://cran.r-project.org/web/packages/hierfstat/index.html.

[51] Kamvar Z.N., Tabima J.F., Grünwald N.J. (2014). *Poppr*: An R Package for Genetic Analysis of Populations with Clonal, Partially Clonal, and/or Sexual Reproduction. PeerJ.

[52] Kamvar Z.N., Brooks J.C., Grünwald N.J. (2015). Novel r Tools for Analysis of Genome-Wide Population Genetic Data with Emphasis on Clonality. Front. Genet..

[53] Dray S., Dufour A. (2007). The Ade4 Package: Implementing the Duality Diagram for Ecologists. J. Stat. Softw..

[54] Bruvo R., Michiels N.K., D’Souza T.G., Schulenburg H. (2004). A Simple Method for the Calculation of Microsatellite Genotype Distances Irrespective of Ploidy Level. Mol. Ecol..

[55] Song X., Yang T., Yan X., Zheng F., Xu X., Zhou C. (2020). Comparison of Microsatellite Distribution Patterns in Twenty-Nine Beetle Genomes. Gene.

[56] Lim L.Y., Ab Majid A.H. (2021). Development and Characterization of Novel Polymorphic Microsatellite Markers for *Tapinoma indicum* (Hymenoptera: Formicidae). J. Insect Sci..

[57] Ding S., Wang S., He K., Jiang M., Li F. (2017). Large-Scale Analysis Reveals That the Genome Features of Simple Sequence Repeats Are Generally Conserved at the Family Level in Insects. BMC Genom..

[58] Nam H.Y., Coates B., Kim K.S., Park M., Lee J.-H. (2015). Characterization of 12 Novel Microsatellite Markers of *Sogatella furcifera* (Hemiptera: Delphacidae) Identified From Next-Generation Sequence Data. J. Insect Sci..

[59] De Meeûs T., Chan C.T., Ludwig J.M., Tsao J.I., Patel J., Bhagatwala J., Beati L. (2021). Deceptive Combined Effects of Short Allele Dominance and Stuttering: An Example with Ixodes Scapularis, the Main Vector of Lyme Disease in the U.S.A. Peer Community J..

[60] Brookes C., Bright J.-A., Harbison S., Buckleton J. (2012). Characterising Stutter in Forensic STR Multiplexes. Forensic Sci. Int. Genet..

[61] Huang C., Ji B., Shi Z., Wang J., Yuan J., Yang P., Xu X., Jing H., Xu L., Fu J. (2025). A Comparative Genomic Analysis at the Chromosomal-Level Reveals Evolutionary Patterns of Aphid Chromosomes. Commun. Biol..

[62] Guichoux E., Lagache L., Wagner S., Chaumeil P., Léger P., Lepais O., Lepoittevin C., Malausa T., Revardel E., Salin F. (2011). Current Trends in Microsatellite Genotyping. Mol. Ecol. Resour..

[63] De Barba M., Miquel C., Lobréaux S., Quenette P.Y., Swenson J.E., Taberlet P. (2016). High-Throughput Microsatellite Genotyping in Ecology: Improved Accuracy, Efficiency, Standardization and Success with Low-Quantity and degradedDNA. Mol. Ecol. Resour..

[64] Lepais O., Chancerel E., Boury C., Salin F., Manicki A., Taillebois L., Dutech C., Aissi A., Bacles C.F.E., Daverat F. (2020). Fast Sequence-Based Microsatellite Genotyping Development Workflow. PeerJ.

[65] Bonin A., Bellemain E., Bronken Eidesen P., Pompanon F., Brochmann C., Taberlet P. (2004). How to Track and Assess Genotyping Errors in Population Genetics Studies. Mol. Ecol..

[66] Hoffman J.I., Amos W. (2004). Microsatellite Genotyping Errors: Detection Approaches, Common Sources and Consequences for Paternal Exclusion. Mol. Ecol..

[67] Koch J.B.U., Branstetter M.G., Cox-Foster D.L., Knoblett J., Lindsay T.-T.T., Pitts-Singer T.L., Rohde A.T., Strange J.P., Tobin K.B. (2023). Novel Microsatellite Markers for *Osmia lignaria* (Hymenoptera: Megachilidae): A North American Pollinator of Agricultural Crops and Wildland Plants. J. Insect Sci..

[68] Wouters R.H.M. (2021). The Genetic Diversity of Natural Populations of *Myzus persicae*, a Polyphagous Aphid Species. Ph.D. Thesis.

[69] Chapuis M.-P., Plantamp C., Streiff R., Blondin L., Piou C. (2015). Microsatellite Evolutionary Rate and Pattern in *Schistocerca gregaria* Inferred from Direct Observation of Germline Mutations. Mol. Ecol..

[70] Rey O., Loiseau A., Facon B., Foucaud J., Orivel J., Cornuet J.-M., Robert S., Dobigny G., Delabie J.H.C., Mariano C.D.S.F. (2011). Meiotic Recombination Dramatically Decreased in Thelytokous Queens of the Little Fire Ant and Their Sexually Produced Workers. Mol. Biol. Evol..

[71] Oldroyd B.P., Allsopp M.H., Gloag R.S., Lim J., Jordan L.A., Beekman M. (2008). Thelytokous Parthenogenesis in Unmated Queen Honeybees (*Apis mellifera capensis*): Central Fusion and High Recombination Rates. Genetics.

[72] Blackman R.L. (1978). Early Development of the Parthenogenetic Egg in Three Species of Aphids (Homoptera: Aphididae). Int. J. Insect Morphol. Embryol..

[73] Srinivasan D.G., Abdelhady A., Stern D.L. (2014). Gene Expression Analysis of Parthenogenetic Embryonic Development of the Pea Aphid, Acyrthosiphon Pisum, Suggests That Aphid Parthenogenesis Evolved from Meiotic Oogenesis. PLoS ONE.

[74] Halkett F., Simon J., Balloux F. (2005). Tackling the Population Genetics of Clonal and Partially Clonal Organisms. Trends Ecol. Evol..

[75] Grünwald N.J., Goodwin S.B., Milgroom M.G., Fry W.E. (2003). Analysis of Genotypic Diversity Data for Populations of Microorganisms. Phytopathology.

[76] Strong W.L. (2016). Biased Richness and Evenness Relationships Within Shannon Wiener Index Values. Ecol. Indic..

