# Supplementary material for: Development of New SSR Markers for High-Throughput Analyses of Peach–Potato Aphid (Myzus persicae Sulzer)
Source: Insects. 2025 Nov 12;16(11):1156. doi: 10.3390/insects16111156 (PMC12653985; doi:10.3390/insects16111156)
Supplement: Supplementary file 1 [file insects-16-01156-s001.zip › insects-3908549-supplementary.pdf]

Development of New SSR Markers for High-Throughput Analyses in Peach-Potato Aphid (*Myzus persicae* Sulzer) - Supplementary material

Jakub Vašek[1], Vladimíra Sedláková[1], Daniela Čílová[1], Martina Melounová[1], Ema Sichingerová[2], Petr Doležal[3], Ervín Hausvater[3] & Petr Sedlák[1]*

[1]Department of Genetics and Breeding, Faculty of Agrobiology Food and Natural Resources, Czech University of Life Sciences Prague, Kamýcká 129, 165 00 Prague, Czech Republic; vasek@af.czu.cz (J.V.); sedlakova@af.czu.cz (V.S.); cilova@af.czu.cz (D.Č.); melounova@af.czu.cz (M.M.)

[2]DIANA Biotechnologies, Průmyslová 596, 252 50 Vestec, Czech Republic; emasichin@gmail.com (E.S.)

[3]Department of Potato Protection, Potato Research Institute Havlíčkův Brod, Ltd., Dobrovského 2366, 580 01 Havlíčkův Brod, Czech Republic; dolezal@vubhb.cz (P.D.); hausvater@vubhb.cz (E.H.)

*Correspondence: sedlak@af.czu.cz

Table S1: Mean error rate per allele (e_a) and per locus (e_l).

**Marker**	e_a [%]	e_l [%]
Myzper-002	0.00	0.00
Myzper-003	0.00	0.00
Myzper-014	0.00	0.00
Myzper-016	0.00	0.00
Myzper-023	0.00	0.00
Myzper-025	0.00	0.00
Myzper-028	0.00	0.00
Myzper-031	0.00	0.00
Myzper-032	0.00	0.00
Myzper-040	0.00	0.00
Myzper-047	0.00	0.00
Myzper-052	11.36	13.64
Myzper-054	0.00	0.00
Myzper-060	2.27	4.55
Myzper-061	0.00	0.00
Myzper-064	0.00	0.00
Myzper-066	0.00	0.00

**Marker**	$\boldsymbol{e_a}$ [%]	$\boldsymbol{e_l}$ [%]
Myzper-071	0.00	0.00
Myzper-073	0.00	0.00
Myzper-095	0.00	0.00
Myzper-106	0.00	0.00
Myzper-112	4.55	4.55
Myzper-121	0.00	0.00
Myzper-131	21.43	42.86
Myzper-137	0.00	0.00
Myzper-146	0.00	0.00
Myzper-148	0.00	0.00
Myzper-154	0.00	0.00
Myzper-162	4.55	4.55
Myzper-166	0.00	0.00
Myzper-170	0.00	0.00
Myzper-172	0.00	0.00
Myzper-174	0.00	0.00
Myzper-180	9.52	14.29
Myzper-182	11.36	22.73
Myzper-191	0.00	0.00
Myzper-195	0.00	0.00
Myzper-197	0.00	0.00
Myzper-200	0.00	0.00
Myzper-207	0.00	0.00
Myzper-208	0.00	0.00
Myzper-209	0.00	0.00
Myzper-212	0.00	0.00
Myzper-214	2.38	4.76
Myzper-215	0.00	0.00
Myzper-231	0.00	0.00
Myzper-244	0.00	0.00
Myzper-257	0.00	0.00
Myzper-268	13.64	13.64

Table S2: SSRs transferability (+) between species.

Assay	Marker	*A. nasturtii*	*A. fabae*	*P. humuli*
1	Myzper-023	+	+	+
1	Myzper-064	-	-	-
1	Myzper-095	-	-	-
1	Myzper-112	-	-	+
1	Myzper-121	-	-	+
1	Myzper-131	-	-	+
1	Myzper-137	-	-	-
1	Myzper-146	-	-	+
1	Myzper-180	-	-	+
1	Myzper-207	-	-	+
1	Myzper-209	-	-	-
1	Myzper-231	-	-	-
2	Myzper-016	-	-	+
2	Myzper-025	-	-	+
2	Myzper-054	-	-	-
2	Myzper-060	-	-	+
2	Myzper-061	-	-	-
2	Myzper-066	-	-	+
2	Myzper-073	-	-	+
2	Myzper-172	-	-	+
2	Myzper-174	-	-	-
2	Myzper-182	-	-	+
2	Myzper-197	-	-	+
2	Myzper-208	-	-	-
2	Myzper-212	-	-	-
3	Myzper-028	-	-	+
3	Myzper-040	+	-	+
3	Myzper-047	-	-	+
3	Myzper-106	-	-	+
3	Myzper-148	-	-	+
3	Myzper-166	-	-	-
3	Myzper-191	-	-	+

Assay	Marker	*A. nasturtii*	*A. fabae*	*P. humuli*
3	Myzper-200	-	-	+
3	Myzper-214	-	-	+
3	Myzper-215	-	-	-
3	Myzper-244	-	-	-
3	Myzper-257	-	-	-
4	Myzper-002	-	-	+
4	Myzper-003	-	-	+
4	Myzper-014	-	-	+
4	Myzper-031	-	-	-
4	Myzper-032	-	-	+
4	Myzper-052	-	-	+
4	Myzper-071	-	-	+
4	Myzper-154	-	+	+
4	Myzper-162	-	-	-
4	Myzper-170	-	-	+
4	Myzper-195	-	-	+
4	Myzper-268	-	-	+

Table S3: Normality and homoskedasticity check for expected heterozygosity (H_{Exp}) and observed heterozy-gosity (H_{Obs}). Statistically significant values are highlighted.

Clone correction	Bartlett's test	Shapiro-Wilk test
BEFORE	$p = 0.0014$	H_{Exp}: p=0.075
		H_{Obs}: p=0.350
AFTER	$p = 0.0077$	H_{Exp}: p=0.093
		H_{Obs}: p=0.442

Table S4: P-values of HWE. Statistically significant values are highlighted.

Marker	$P_{\chi 2\text{-test}}$ [a]	$P_{exact\text{-test}}$ [b]	$P_{\chi 2\text{-test}}$ [c]	$P_{exact\text{-test}}$ [d]
Myzper-023	0.01454	0.004	0.4689	0.5758
Myzper-064	0	0	3.208e-09	0
Myzper-095	0	0	0	0
Myzper-112	0.3649	0.645	0.7856	1
Myzper-121	0.6085	0.3542	0.9535	0.7439

Marker	$P_{\chi 2\text{-test}}$ [a]	$P_{\text{exact-test}}$ [b]	$P_{\chi 2\text{-test}}$ [c]	$P_{\text{exact-test}}$ [d]
Myzper-137	0	0	0	0
Myzper-146	5.498e-11	0	0.0002308	4e-04
Myzper-209	0	0	3.117e-06	1e-04
Myzper-231	0.8772	0.5549	0.2874	0.1841
Myzper-016	0	0	6.858e-06	0
Myzper-025	0	0	0.0005596	3e-04
Myzper-054	3.331e-16	0	0.2714	0.308
Myzper-060	0	0	4.989e-05	0
Myzper-061	0	0	1.823e-11	0
Myzper-066	7.327e-13	0	0.01909	0.0094
Myzper-073	0.0001939	0	0.1924	0.1088
Myzper-172	1.819e-09	0	0.0008479	0.0011
Myzper-174	0	0	0	0
Myzper-197	0	0	4.001e-05	0
Myzper-208	3.672e-13	0	0.005042	0.0058
Myzper-212	0	0	5.717e-05	0
Myzper-028	0	0	0.07708	0
Myzper-040	0	0	0	0
Myzper-047	0	0	2.322e-08	0
Myzper-106	0	0	2.22e-16	0
Myzper-148	0.001837	0.0038	0.5238	0.707
Myzper-166	0	0	1.533e-07	0
Myzper-191	0	0	0.0001919	0
Myzper-200	0	0	2.955e-13	4e-04
Myzper-214	0	0	6.419e-10	0
Myzper-215	0	0	0.006445	2e-04
Myzper-244	1.603e-06	0	0.2857	0.208
Myzper-257	4.33e-14	0	0.05781	0.0798
Myzper-002	0	0	3.151e-05	1e-04
Myzper-003	0	0	1.587e-10	0
Myzper-014	1.456e-06	0	0.09666	0.1964
Myzper-031	0	0	0	0
Myzper-032	0.02299	0.0269	0.786	0.7697
Myzper-052	0	0	0	0
Myzper-071	0	0	5.203e-06	0

Marker	$P_{\chi 2\text{-test}}$ [a]	$P_{\text{exact-test}}$ [b]	$P_{\chi 2\text{-test}}$ [c]	$P_{\text{exact-test}}$ [d]
Myzper-154	0	0	0.0003929	7e-04
Myzper-162	0	0	2.37e-05	0
Myzper-170	1.554e-15	0	0.2424	0.2138
Myzper-195	0	0	2.317e-08	0
Myzper-268	0.9797	1	0.9948	1

Note: [a]p-value for χ^2-test before clone correction, [b]p-value for exact test before clone correction, [c]p-value for χ^2-test after clone correction, [d]p-value for exact test after clone correction.

Table S5: Population descriptors for each PCoA cluster before and after clone correction part 1 - observed heterozygosity (H_{Obs}), expected heterozygosity (H_{Exp}), inbreeding coefficient (F_{IS}).

		N		H_{Obs}		H_{Exp}		F_{IS}	
**PCoA**	**Cluster**	**before**	**after**	**before**	**after**	**before**	**after**	**before**	**after**
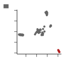	BR	48	10	0.5315	0.5244	0.2697	0.2813	-0.9914	-0.9603
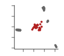	C	77	38	0.5692	0.5452	0.4724	0.4635	-0.2064	-0.1792
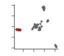	CL	143	24	0.5748	0.5604	0.2906	0.2987	-0.9849	-0.9134
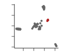	CR	37	6	0.4877	0.4896	0.2496	0.2780	-0.9808	-0.9104
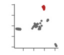	TR	60	19	0.5026	0.4922	0.2584	0.2643	-0.9606	-0.9090
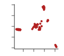	Total	365	97	0.5468	0.5332	0.4609	0.4682	-0.1868	-0.1396

Table S6: Population descriptors for each PCoA cluster before and after clone correction part 2 - Shannon-Weaver index (H), Taylor's index (G), Simpson's diversity index (λ).

		H		G		λ	
**PCoA**	**Cluster**	**before**	**after**	**before**	**after**	**before**	**after**
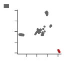	BR	0.8946	2.3026	1.5059	10	0.3359	0.9000
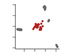	C	2.8481	3.6376	7.3836	38	0.8646	0.9737
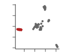	CL	1.1489	3.1781	1.6200	24	0.3827	0.9583
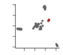	CR	0.9252	1.7918	1.7049	6	0.4134	0.8333
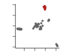	TR	1.8608	2.9444	2.9703	19	0.6633	0.9474

PCoA	Cluster	H before	H after	G before	G after	λ before	λ after
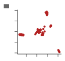	Total	3.0593	4.5747	7.8502	97	0.8726	0.9897

Table S7: Population descriptors for each PCoA cluster before and after clone correction part 3 - evenness (E) and standardized association index ($\bar{r}_D$).

PCoA	Cluster	E before	E after	r_D before	r_D after
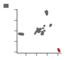	BR	0.3498	1	0.1113	0.0000
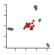	C	0.3927	1	0.4281	0.2044
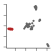	CL	0.2877	1	0.0265	-0.0348
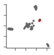	CR	0.4630	1	-0.0392	-0.1454
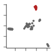	TR	0.3629	1	0.0277	-0.0049
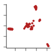	Total	0.3372	1	0.3652	0.2212

Table S8: Pairwise F_{ST} values before (blue) and after (red) clone correction.

Cluster	C	CL	BR	TR	CR
**C**		0.1815	0.3746	0.2759	0.2743
**CL**	0.1557		0.5533	0.4015	0.4553
**BR**	0.3540	0.5355		0.5186	0.5133
**TR**	0.2578	0.3917	0.5017		0.3781
**CR**	0.2281	0.4213	0.4762	0.3470	

Note: Shortcuts refer to approximate position of identified clusters by PCoA. BR = bottom right, C = center, CL = center left, CR = center right, TR = top right.

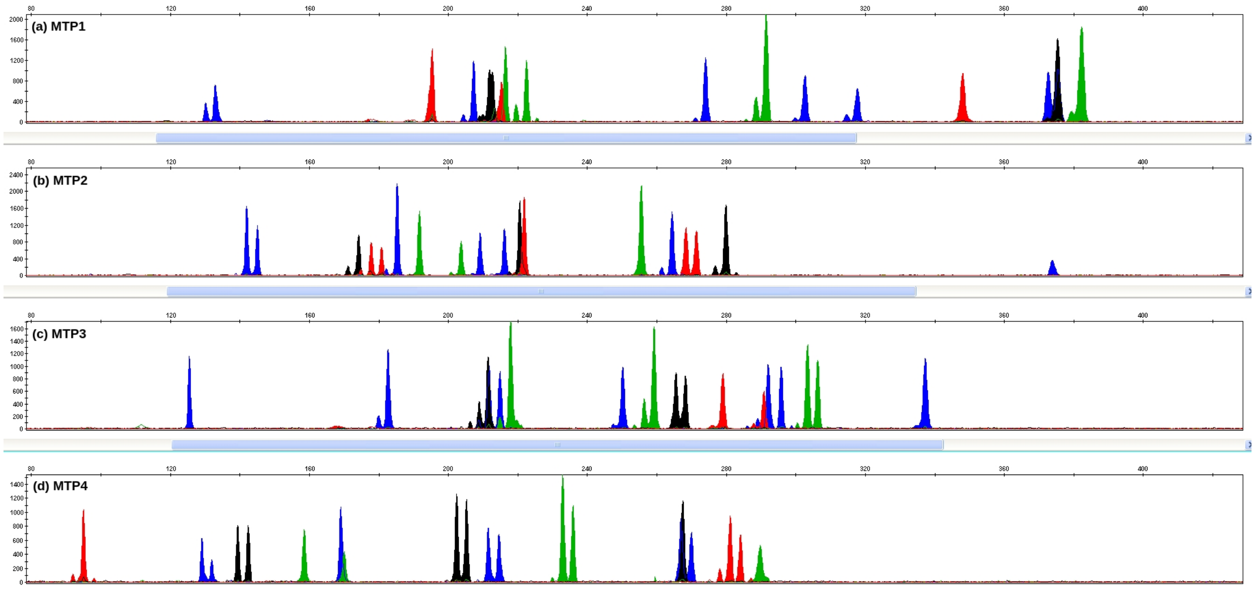

Figure S1: Initial allelic ladder. Multiplex assay 1 - 4 (a-d).

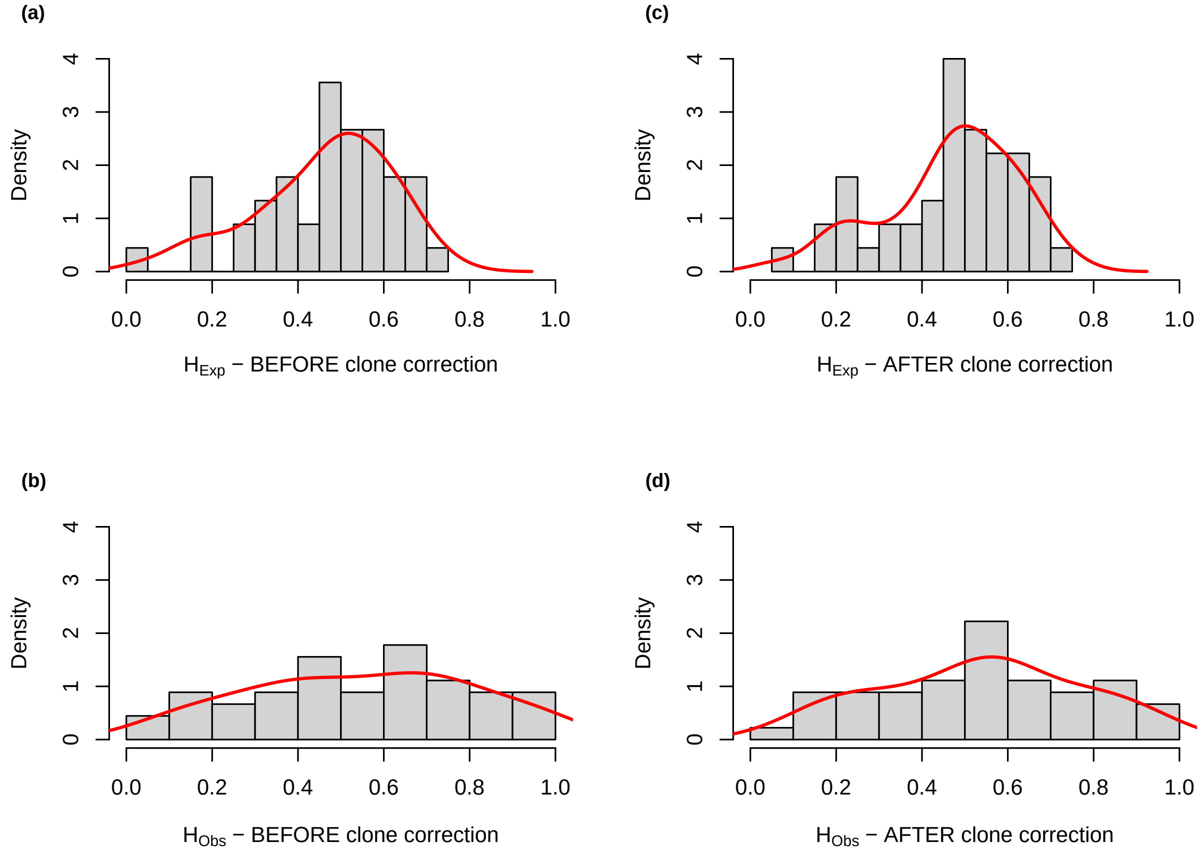

Figure S2: Exploratory data analysis of expected heterozygosity (H_{Exp}) before (a) and after (c) clone correction and observed heterozygosity (H_{Obs}) before (b) and after (d) clone correction.

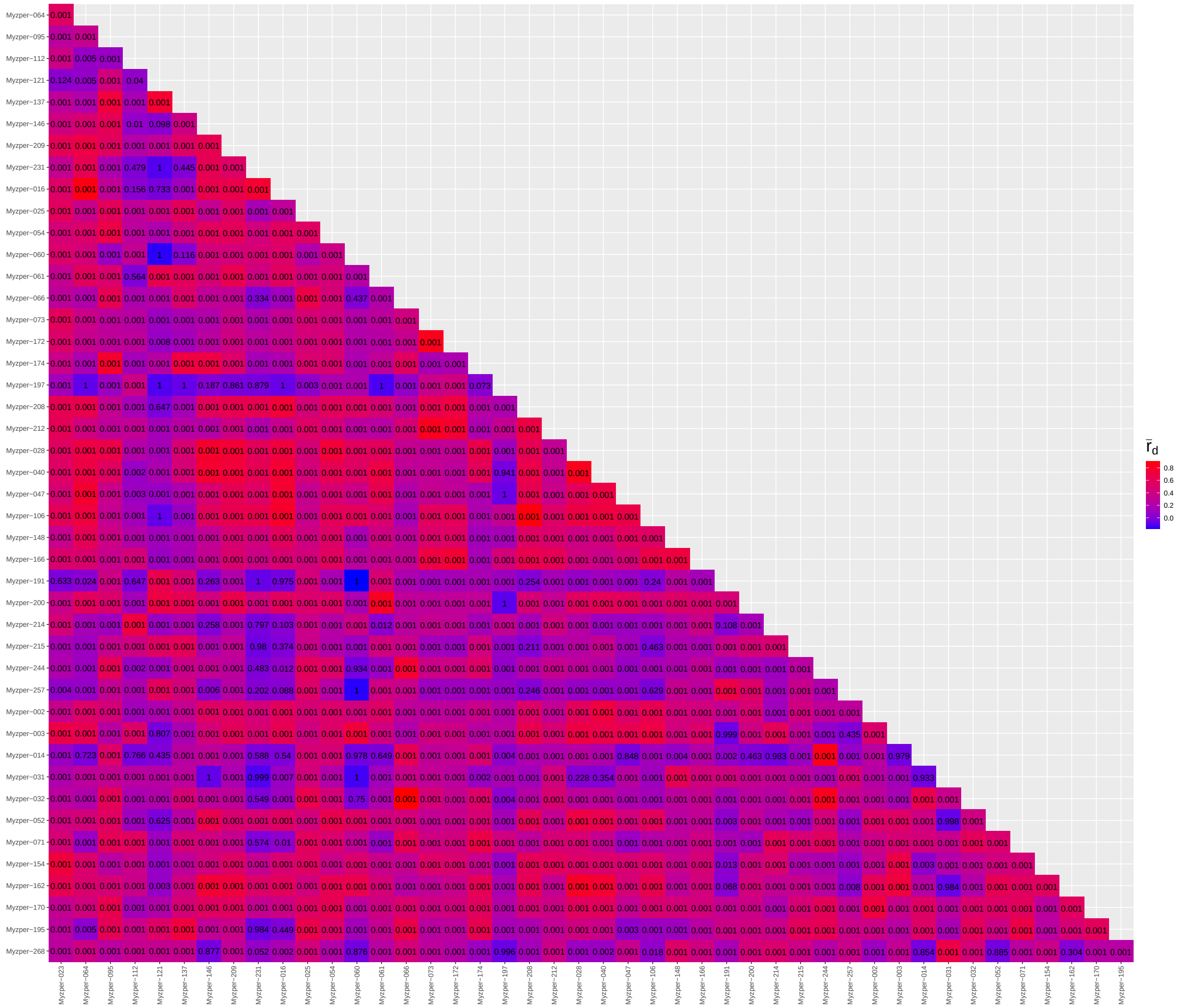

Figure S3: Paired $\bar{r}_D$ before clone correction. Depicted values are p-values for given pair of loci. Color heatmap reflects degree of association between loci.

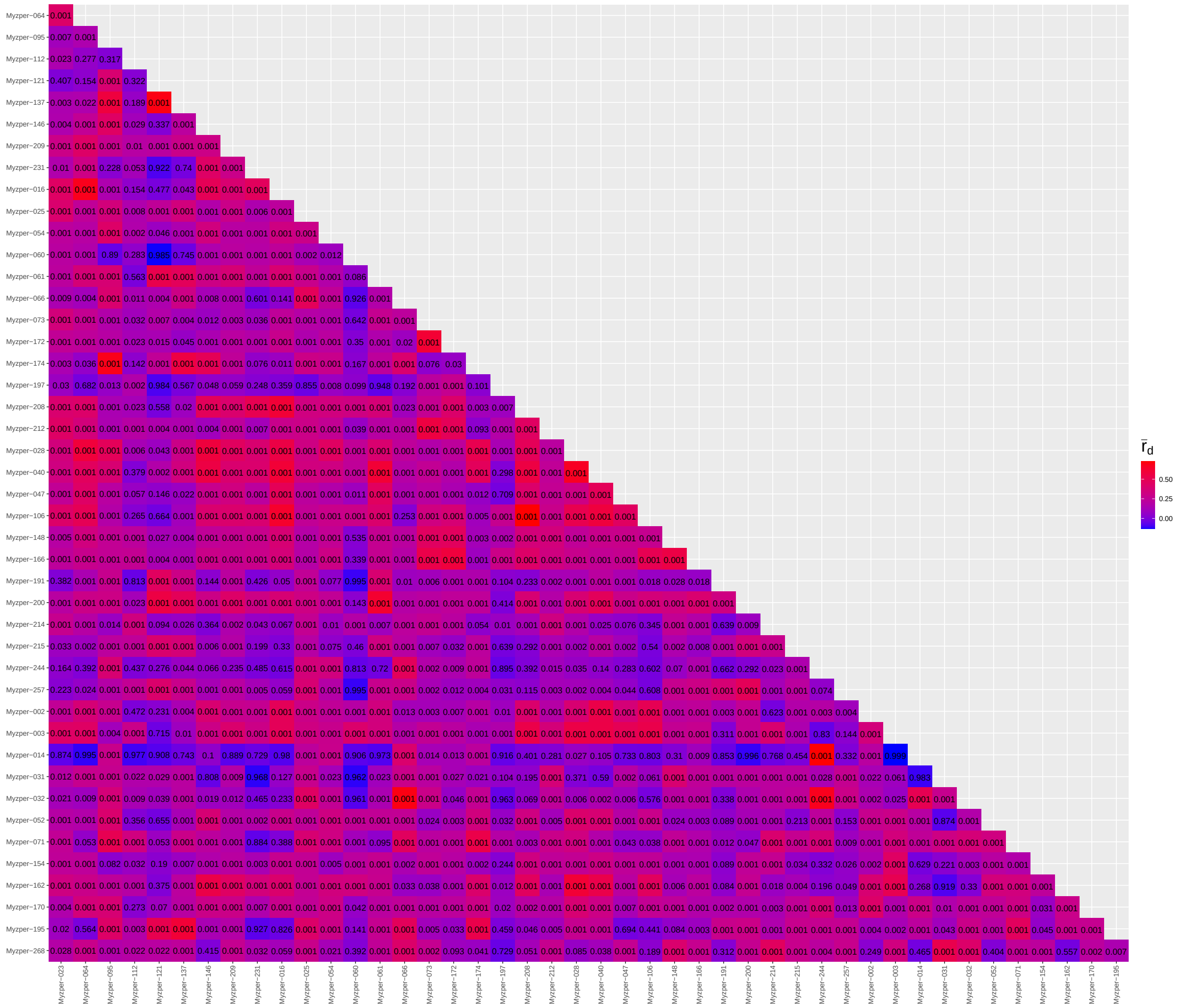

Figure S4: Paired $\bar{r}_D$ after clone correction. Depicted values are p-values for given pair of loci. Color heatmap reflects degree of association between loci.

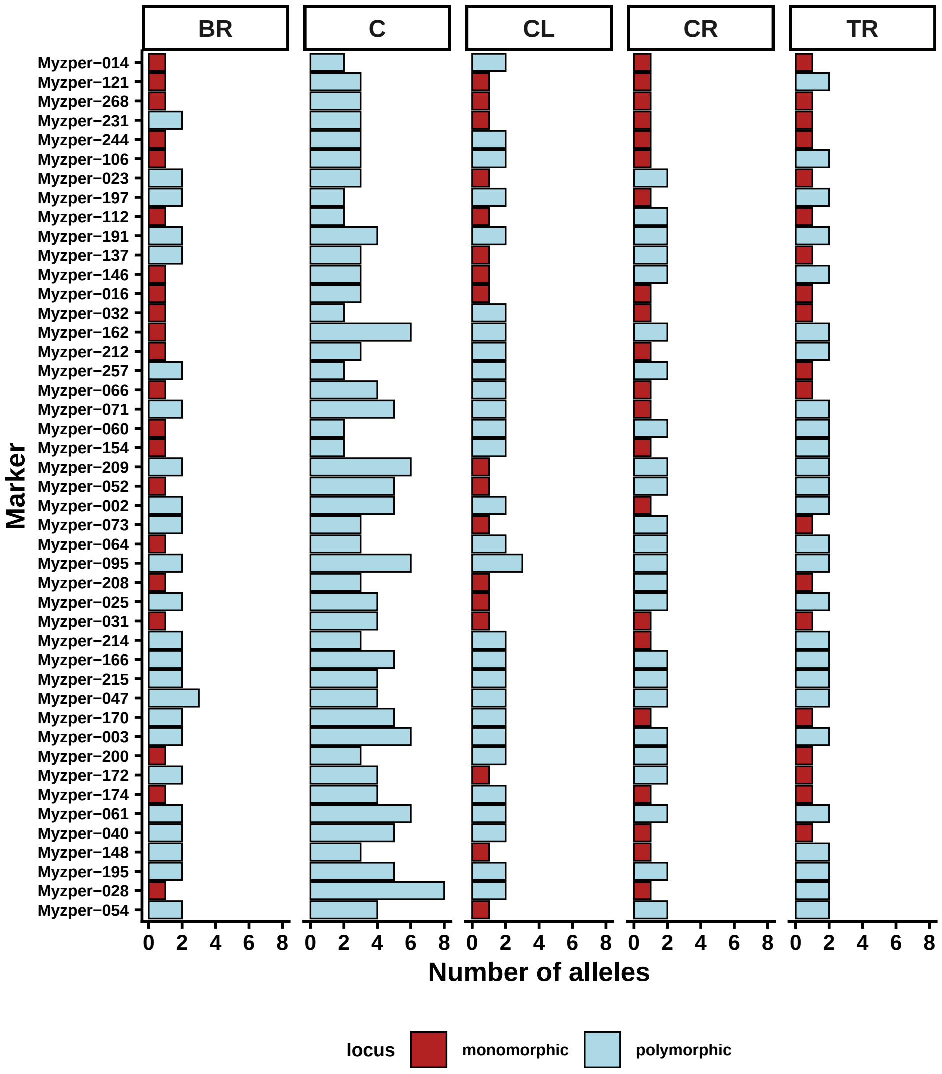

Figure S5: Number of alleles per locus per PCoA cluster. Shortcuts refer to approximate position of identified clusters by PCoA. BR = bottom right, C = center, CL = center left, CR = center right, TR = top right.

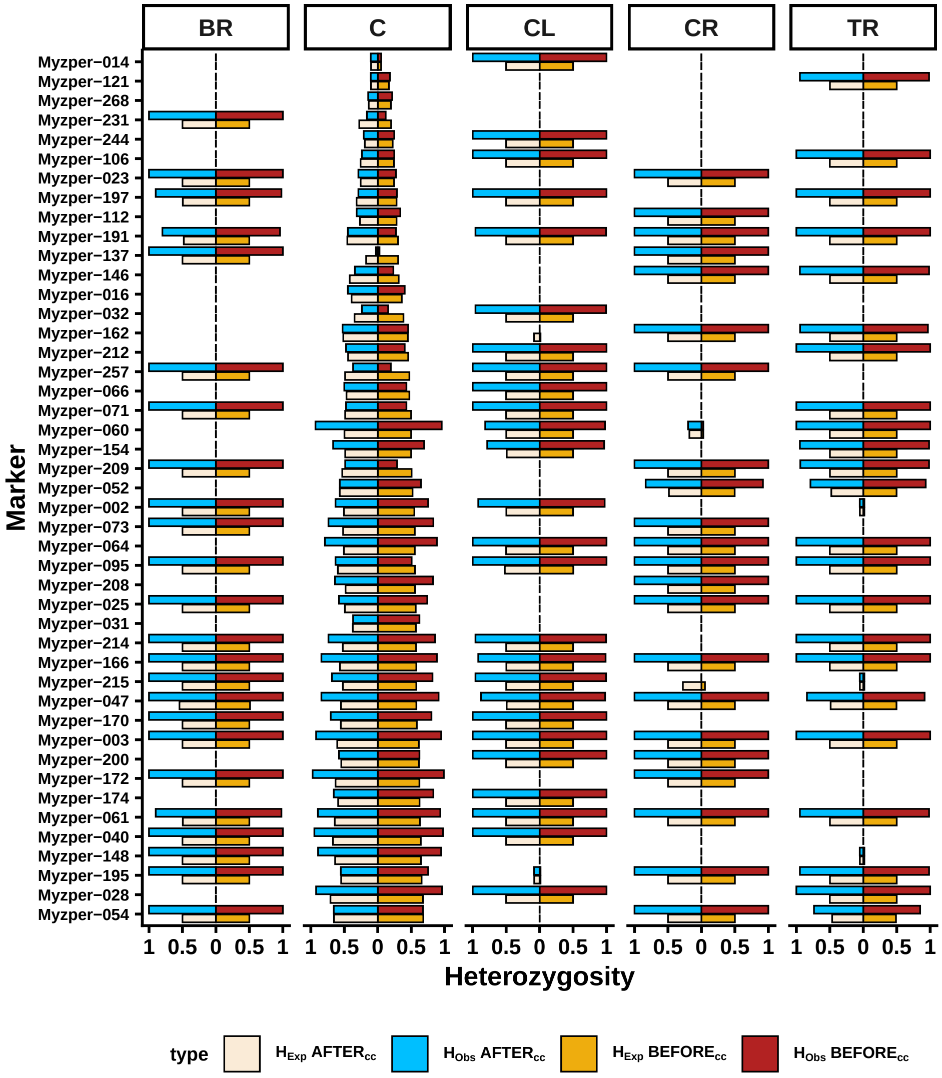

Figure S6: Observed (H_{Obs}) and expected (H_{Exp}) heterozygosity per cluster identified by PCoA. Shortcuts refer to approximate position of identified clusters by PCoA. BR = bottom right, C = center, CL = center left, CR = center right, TR = top right.

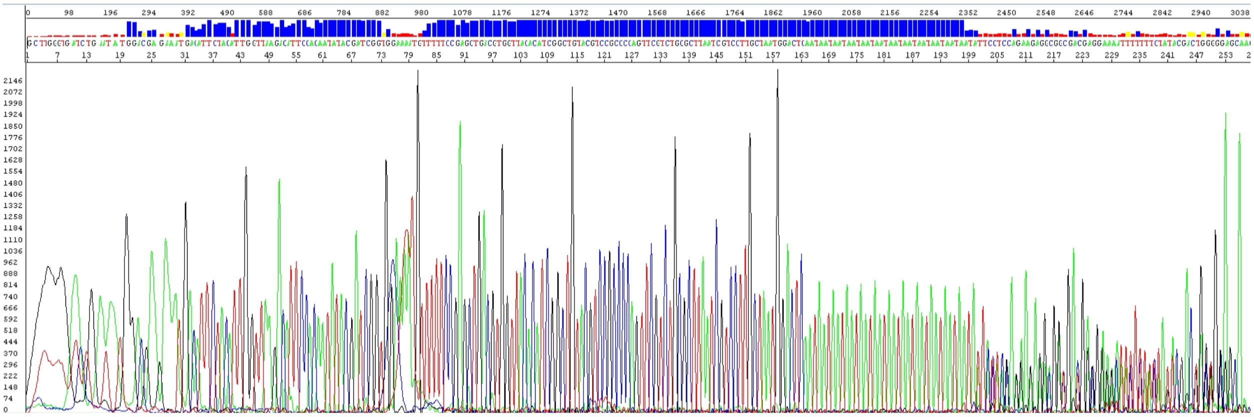

Figure S7: Example of Sanger sequencing with ambiguous sequence signal.

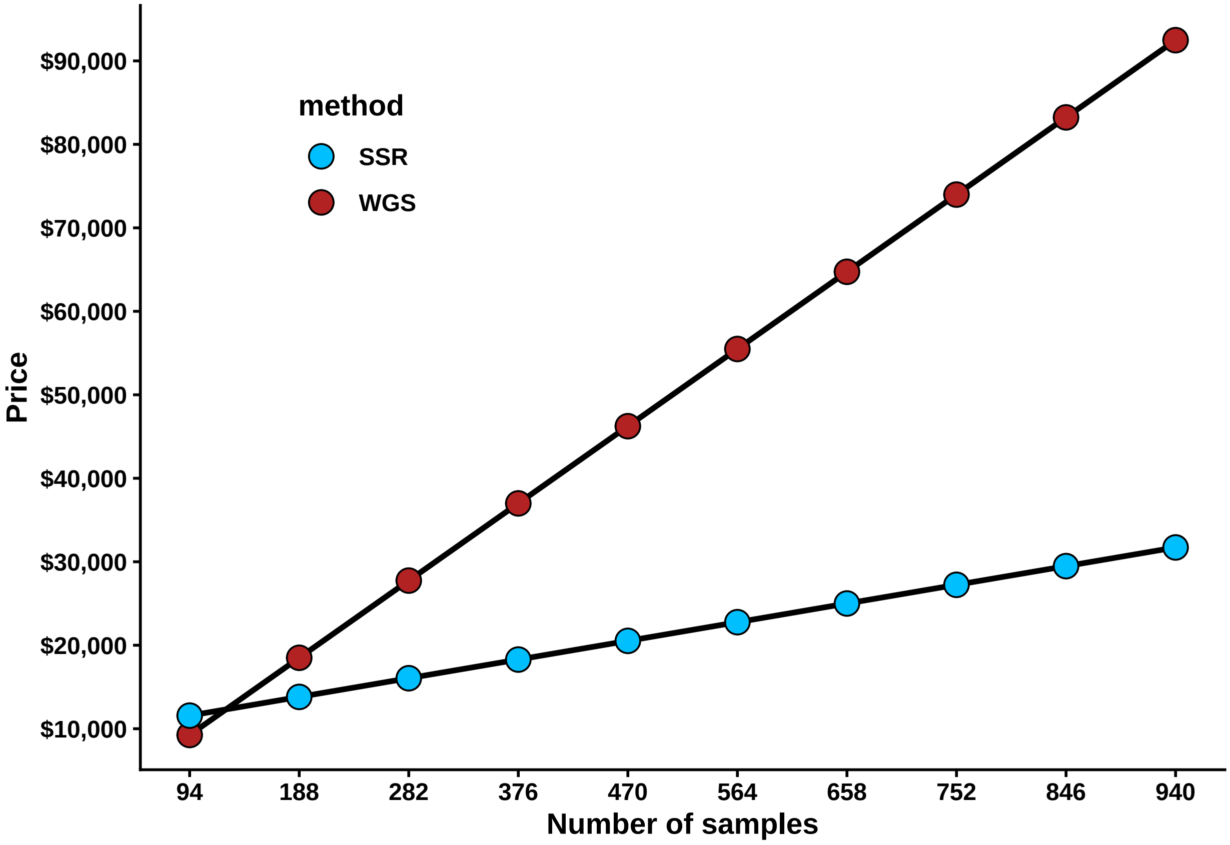

Figure S8: Estimated price of whole genome sequencing (WGS) and SSR analysis. Rough estima-tion of costs per common sample set (1 set = 94 samples). Initial parameters for WGS as a service are ~10x coverage, approx. 375 Gb of raw data, without bioinformatic service. Price per library and sequencing costs were adopted from pricelist of local sequencing service provider.
